# Genome streamlining of *Pseudomonas putida* B6-2 for bioremediation

**DOI:** 10.1128/msystems.00845-24

**Published:** 2024-11-12

**Authors:** Siqing Fan, Hao Ren, Xueni Fu, Xiangyu Kong, Hao Wu, Zhenmei Lu

**Affiliations:** 1 MOE Laboratory of Biosystem Homeostasis and Protection, College of Life Sciences, Zhejiang University, Hangzhou, China; 2 Cancer Center, Zhejiang University, Hangzhou, China; University of California, San Diego, La Jolla, California, USA

**Keywords:** *Pseudomonas putida*, genome streamlining, genome-scale metabolic models (GEMs), stress tolerance, environmental remediation

## Abstract

Microbial transformation is a favored approach for environmental remediation. However, the effectiveness of microbial remediation has been limited by the lack of chassis cells with satisfactory contaminant degradation performance. *Pseudomonas putida* B6-2, with a wide substrate spectrum and high solvent tolerance, is a chassis strain with great potential for application in environmental remediation. Here, guided by bioinformatic analyses and genome-scale metabolic model (GEM) predictions, we successfully optimized *P. putida* B6-2 by rationally reducing its nonessential genetic components and generating a more robust genome-streamlined strain, *P. putida* BGR4. Several improvements were observed compared with the original *P. putida* B6-2 strain, including a 1.4 × 10^5^-fold increase in electroporation efficiency, an 8.3-fold increase in conjugation efficiency, improved glycerol utilization capability, and increased phenol utilization after heterologous expression of the phenol monooxygenase encoded by *dmpKLMNOP*. Additionally, *P. putida* BGR4 exhibited enhanced tolerance to several stressors, including starvation, oxidative stress, and DNA damage. Transcriptomic analysis revealed that genome streamlining led to the upregulation of genes involved in the “carbon metabolism” and “tricarboxylic acid cycle” pathways in *P. putida* BGR4, which likely contributed to the superior phenotype of *P. putida* BGR4 in terms of carbon source utilization and contaminant degradation capabilities. Furthermore, the absence of four prophages was identified as a potential cause of the enhanced stress resistance observed in *P. putida* BGR4. Overall, we developed a combined genome-streamlining strategy involving bioinformatic analyses and GEM predictions and generated a more robust chassis strain, *P. putida* BGR4, which expands the repertoire of chassis cells for environmental remediation.

**IMPORTANCE:**

Despite the development of many chassis cells, there is still a lack of robust chassis cells with satisfactory contaminant degradation performance. Targeted genome streamlining is an effective way to provide powerful chassis cells. However, genome streamlining does not always lead to the improved phenotypes of genome-streamlined chassis cells. In this research, a novel procedure that combined bioinformatic analyses and GEM predictions was proposed to guide genome streamlining and predict the effects of genome streamlining. This genome streamlining procedure was successfully applied to *Pseudomonas putida* B6-2, which was a chassis cell with great potential for application in environmental remediation and resulted in the generation of a more robust chassis cell, *P. putida* BGR4, thereby providing a superior chassis cell for efficient and sustainable environmental remediation and a valuable framework for guiding the genome streamlining of strains for other applications.

## INTRODUCTION

With the rapid development of industry and agriculture, an increasing number and variety of refractory contaminants are being produced every year, posing a major threat to public health (1). Strategies to degrade these refractory contaminants have received widespread attention. Advantageous properties, such as sustainability and environmental friendliness, have made bacteria favorable candidates for environmental remediation. However, most microbes that contain only the catabolic genes for a single compound cannot be effectively used in most pollution scenarios involving multiple refractory contaminants (2). Artificial combinations of microorganisms with different abilities to degrade refractory contaminants may solve this problem, but negative interactions between the components of the microbial consortium would disturb the microbial community and limit the contaminant degradation efficiency of the consortium (3).

The emergence and rapid development of synthetic biology has made it possible to construct robust chassis cells with combined pollution degradation capabilities. Several existing natural contaminant degradation pathways can be recruited together and assembled in the chassis cells by molecular biological techniques to produce functional synthetic pathways, thus expanding the substrate range of the chassis cells and providing a favorable solution for combined pollution remediation (4). To date, there are only limited reports on the construction of chassis cells for the remediation of combined pollution using synthetic biology (2). *Pseudomonas* strains are model organisms that are endowed with metabolic, physiological, and stress endurance traits that could help them fulfill the demands of combined pollution remediation (5), and *Pseudomonas putida* KT2440 is the most commonly used chassis strain in such studies (6–8). However, as a chassis strain for combined pollution degradation, *P. putida* KT2440 has certain limitations, as it can degrade only a limited number of simple aromatic compounds (9) and cannot degrade refractory contaminants such as polycyclic aromatic hydrocarbons (PAHs) and dioxin analogs, which are toxic and pose serious health risks (10, 11). Therefore, it is necessary to develop new chassis cells with enhanced contaminant degradation capabilities.

*P. putida* B6-2 was a “superstar” for the mineralization of PAHs and dioxin-like compounds (12). Compared with *P. putida* KT2440 (Table S1), *P. putida* B6-2 possesses several advantageous traits that make it more suitable for bioremediation, such as a wide substrate spectrum, diverse contaminant degradation pathways, and strong solvent tolerance (12–14). Therefore, we expect *P. putida* B6-2 to be a promising chassis strain with significant potential for combined pollution remediation. However, during the process of using *P. putida* B6-2 as a chassis strain for heterologous expression of functional contaminant degradation pathways to enable the remediation of more refractory contaminants, it was observed that the transformation efficiency of *P. putida* B6-2 was remarkably low. This limitation prevents *P. putida* B6-2 from expressing functional contaminant degradation pathways and degrading a broader range of contaminants, thereby hindering its broader application for combined pollution remediation.

Targeted genome streamlining has been proven to be an effective approach for enhancing the gene editing efficiency of various organisms such as *Escherichia coli* (15), *P. putida* (16), and *Schlegelella brevitalea* (17). Moreover, targeted genome streamlining has also led to several enhanced characteristics in genome-streamlined chassis cells, such as increased growth rates and biomass yield (15, 18, 19), enhanced fitness under stressful conditions (20, 21), and boosted heterologous gene expression (16, 18, 21, 22). Therefore, we aimed to optimize *P. putida* B6-2 by targeted genome streamlining to address its limitations and further enhance its capabilities, thereby obtaining a more resilient and efficient chassis cell for environmental remediation.

During the process of targeted genome streamlining, various bioinformatic methods are employed to analyze genomes for redundant components, such as prophages (18, 20), genomic islands (GIs) (16, 17), and other mobile genetic elements (15, 23). These redundant components are then sequentially eliminated. However, the outcomes of genome streamlining have been unpredictable. Even if the elimination of a single fragment could lead to an improved phenotype, the successive elimination of multiple fragments might also yield an unpredictable phenotype, such as decreased growth (19, 24) and reduced production (16, 25). Moreover, the specific mechanisms underlying phenotypic changes after genome streamlining have not been fully characterized. Genome streamlining inherently involves uncertainty, underscoring the need for a tool capable of accurately predicting the effects of genome streamlining on strains.

Genome-scale metabolic models (GEMs) are chemical stoichiometric matrices generated by linking mass-balanced metabolic reactions with gene-protein-reaction associations and provide a comprehensive numerical summary of whole-cell biochemistry at the system level (26, 27). GEMs have been successfully applied to increase biosynthesis yields (28, 29) and bioremediation efficiencies (26, 30). However, to our knowledge, there are no reports to date on the use of GEMs for the rational targeted genome-streamlining strategies (31). Flux balance analysis (FBA) is a widely used biochemical network analysis method (32). By selecting a specific objective function (such as biomass) in GEMs for FBA, it is feasible to rapidly predict the influence of genome streamlining on the target traits of genome-streamlined strains. This approach allows the simulation and preliminary forecasting of the impacts of different genome-streamlining strategies on target traits within GEMs to prevent “blind” genome streamlining.

**Fig 1:**
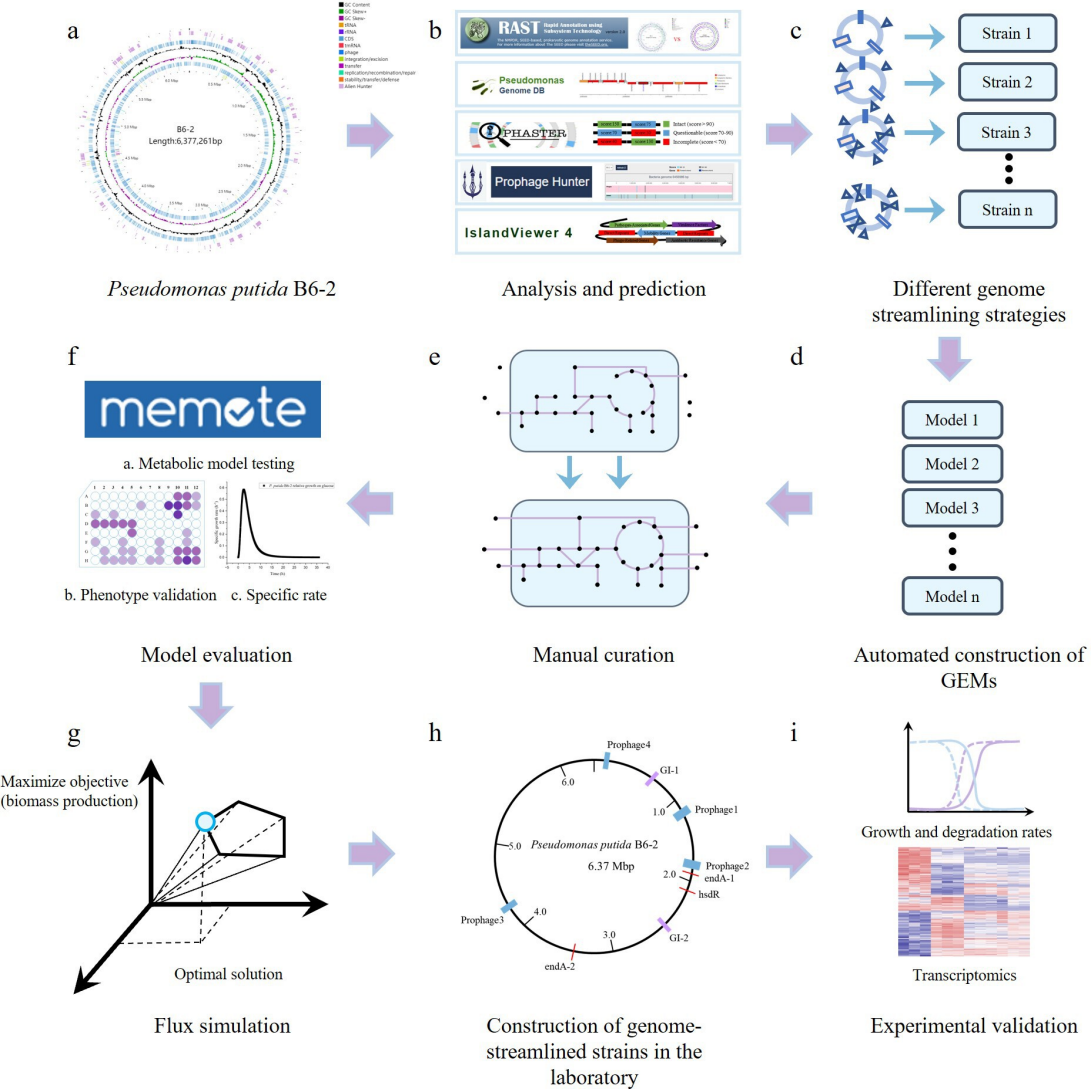
Schematic of the genome-streamlining workflow based on bioinformatic analyses and GEM predictions. (**a**) The genome of *P. putida* B6-2. (**b**) Various bioinformatic analysis and prediction methods were used to identify the fragments to be deleted. (**c**) Several genome-streamlined strains were constructed based on different genome-streamlining strategies. (**d**) Draft GEMs were then generated by using an automated modeling tool called gapseq (33). (**e**) Several manual curation steps (including gap filling, model revision, and parameter calibration) were conducted to improve the quality of the GEMs, and more details about manual curation steps could be found in Materials and Methods. (**f**) Memote (34) was used to score the GEMs, and the qualitative (such as predicting the ability of strains to utilize carbon sources) and quantitative (such as predicting the maximum growth rates [MGRs] of strains) predictive abilities of the GEMs were tested. (**g**) The GEMs were subjected to FBA in MATLAB using the COBRA Toolbox v.3.0 (35). (**h**) The genome-streamlined strains were constructed *in vivo* with the optimal genome-streamlining strategy. (**i**) The target traits were evaluated, and changes in transcription levels between *P. putida* B6-2 and *P. putida* BGR4 were tracked via transcriptomic analysis.

Therefore, in this study, we systematically eliminated redundant elements within the *P. putida* B6-2 genome based on bioinformatic analyses and GEM predictions, aiming to construct an optimal chassis cell for environmental remediation (Fig. 1). Furthermore, the electroporation and conjugation efficiencies, growth characteristics, contaminant degradation capabilities, and stress resistance of *P. putida* B6-2 and its genome-streamlined strains were evaluated to systematically characterize the effects of genome streamlining. Finally, a transcriptomic analysis of *P. putida* B6-2 and *P. putida* BGR4 was conducted to provide valuable insights into the mechanisms underlying the improved phenotypes of the genome-streamlined strains.

## RESULTS

### Determination of deletion targets

The presence of prophages and GIs in the genome of a chassis cell is redundant, as their replication and expression consume the energy of the chassis cell, while the presence of redundant nucleases also hampers the gene editing efficiency of the chassis cell (16, 18, 21). Therefore, prophages, GIs, and redundant nuclease-encoding genes were targeted for deletion. Specifically, PHASTER (36) and Prophage Hunter (37) were used to predict prophages, while IslandViewer 4 (38) was utilized to analyze GIs. Additionally, the genome sequences were subjected to annotation with the RAST server (39) and the Pseudomonas Genome Database (40) to identify redundant nuclease-encoding genes. The identified deletion regions are detailed in Table S2. Overall, four prophages, two GIs and three nuclease-encoding genes were deleted, for a total size of 259.7 kb, accounting for 4.1% of the *P. putida* B6-2 genome.

### Construction and evaluation of the GEMs

**Fig 2:**
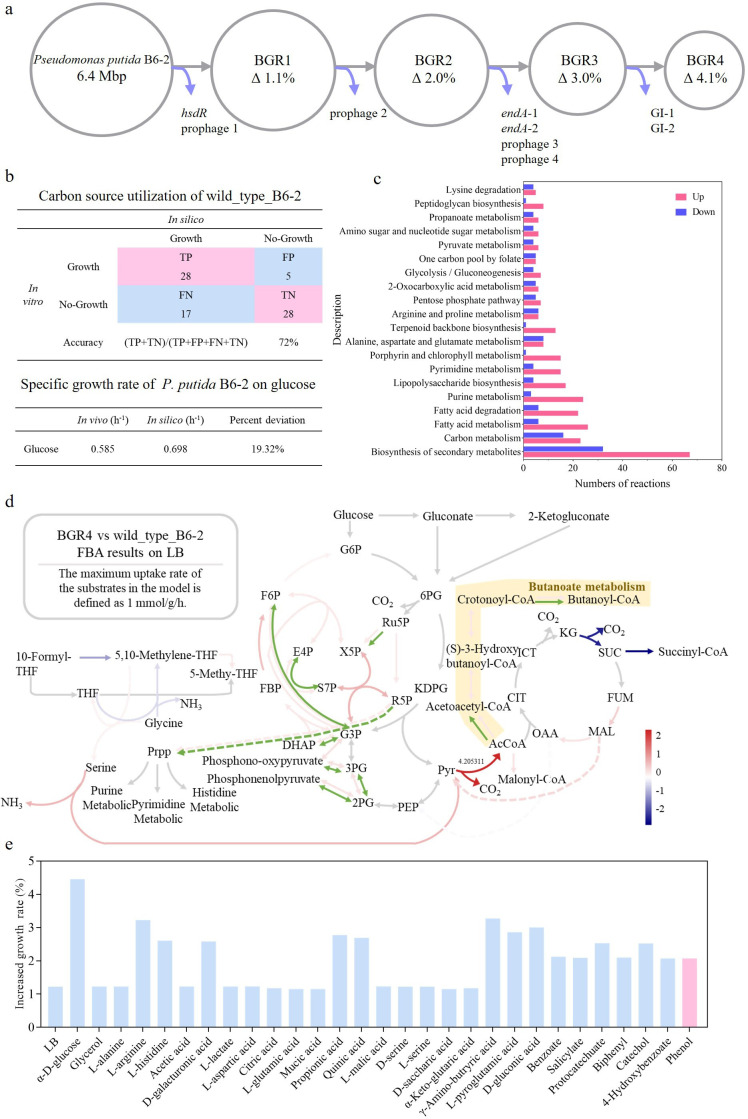
Construction of genome-streamlined strains and GEM-predicted results. (**a**) A pipeline for the construction of genome-streamlined strains. (**b**) Results of the wild_type_B6-2 model’s prediction of the carbon source utilization capability of *P. putida* B6-2 and its ability to quantitatively predict the MGR of *P. putida* B6-2 on glucose. TP, ture positive; FP, false positive; FN, false negative; TN, ture nagetive. (**c**) KEGG enrichment analysis results of differential flux reactions between model wild_type_B6-2 and model BGR4 after FBA on LB. (**d**) Differential flux reactions enriched in the “carbon metabolism” pathway in FBA results and strictly constrained reactions enriched in the “carbon metabolism” pathway in flux variability analysis (FVA) results of model BGR4 and model wild_type_B6-2 on LB. The green arrows represent reactions that were strictly constrained in FVA results. The gray arrows indicate that there was no significant difference in FBA results. G6P, glucose-6-phosphate; F6P, fructose-6-phosphate; FBP, fructose-1,6-bisphosphate; DHAP, dihydroxyacetone phosphate; 6PG, 6-phosphogluconate; KDPG, 2-keto-3-deoxy-6-phosphogluconate; Ru5P, ribulose-5-phosphate; R5P, ribose-5-phosphate; X5P, xylulose-5-phosphate; S7P, sedoheptulose-7-phosphate; E4P, erythrose-4-phosphate; G3P, glyceraldehyde-3-phosphate; 3PG, 3-phosphoglycerate; 2PG, 2-phosphoglycerate; PEP, phosphoenolpyruvate; Pyr, pyruvate; AcCoA, acetyl-coenzyme A; OAA, oxaloacetate; CIT, citrate; ICT, isocitrate; KG: 2-oxo-glutarate; SUC, succinate; FUM, fumarate; MAL, malate; Prpp, 5-phospho-alpha-D-ribose 1-diphosphate. (**e**) The enhanced MGRs of *P. putida* BGR4 in the presence of LB, 28 positive carbon sources, and phenol (shown in pink) compared with those of *P. putida* B6-2 predicted by GEMs.

Once the deletion targets were designed, they were divided into four portions, with each portion comprising approximately 1% of the total genome content. Subsequently, four genome-streamlined strains, designated *P. putida* BGR1, *P. putida* BGR2, *P. putida* BGR3, and *P. putida* BGR4, were designed based on the schematic diagram shown in Fig. 2a. The GEMs for these strains were then automatically constructed using the gapseq tool; detailed information about the GEMs is shown in Table S3.

The quality of the GEMs constructed by gapseq was assessed using the model wild_type_B6-2 as an example; this model received a score of 85 in the Memote report (File S4) and achieved an accuracy ratio of 72% in predicting the utilization of 78 carbon sources by *P. putida* B6-2 in the carbon source utilization capacity experiment (Fig. 2b; Tables S4 and S5). Subsequently, the model wild_type_B6-2 was used to predict the maximum growth rate (MGR) of *P. putida* B6-2 on glucose to assess its quantitative predictive capability, and the difference between the *in silico* prediction and the *in vitro* measurements was lower than 20%, indicating that the model wild_type_B6-2 has reliable quantitative predictive capability (Fig. 2b). The above results indicated that all GEM quality validation experiments consistently demonstrated that the model wild_type_B6-2 constructed by gapseq was reliable.

### The GEM-predicted results showed that, compared with *P. putida* B6-2, *P. putida* BGR4 exhibited higher MGRs on all the tested carbon sources

Once GEMs of reliable quality were obtained, FBA was performed in MATLAB to predict the effect of genome streamlining on target traits. For the chassis cells, higher growth rates indicated that the chassis cells could utilize carbon sources more efficiently for growth, allowing faster acquisition and utilization of resources and gaining competitive advantages. Therefore, growth rate was chosen as the target trait in this study. Then, biomass was set as the objective function to solve for the MGRs on LB (default gap-filling medium) and 28 positive carbon sources (from the carbon source utilization capacity experiment) in the GEMs. The GEM-predicted results showed that genome streamlining did not reduce but rather slightly increased the MGRs of the genome-streamlined strains (Table S6). Moreover, compared with *P. putida* B6-2 and the other genome-streamlined strains, *P. putida* BGR4 had the highest MGRs in the presence of LB and 28 positive carbon sources. The model wild_type_B6-2 and model BGR4 were then subjected to FBA on LB to figure out the reason why the MGR of *P. putida* BGR4 was higher. KEGG enrichment results showed that differential flux reactions were mainly enriched in pathways such as “biosynthesis of secondary metabolites,” “carbon metabolism,” and “fatty acid metabolism” (Fig. 2c). Further analysis of the “carbon metabolism” pathway suggested that the higher MGR of model BGR4 on LB might be attributed to enhanced pathways such as the “pentose phosphate (PP) pathway,” “Embden-Meyerhof-Parnas (EMP) pathway,” and “butanoate metabolism” pathway (Fig. 2d). Furthermore, flux variability analysis (FVA) was used to help identify which reactions were strictly constrained (with nearly fixed fluxes) under conditions of maximal growth rate. The results indicated that nine reactions in the “carbon metabolism” pathway were strictly constrained, which were also enriched in the “PP pathway,” the “EMP pathway,” and “butanoate metabolism” pathway (Fig. 2d).

The ability to heterologously express contaminant degradation gene clusters is crucial for chassis cells for combined pollution remediation purposes. Therefore, by incorporating the phenol transport reaction and the phenol hydroxylation reaction into the GEMs, the MGRs of *P. putida* B6-2 and its genome-streamlined strains on phenol were also predicted. The prediction results showed that *P. putida* BGR4 still had the highest MGR on phenol (Table S6). In summary, compared with *P. putida* B6-2, *P. putida* BGR4 had enhanced MGRs in the presence of LB, 28 positive carbon sources, and phenol, with improvements ranging from 1.15% to 4.46% (Fig. 2e). Consequently, the genome-streamlining strategy for *P. putida* BGR4 was adopted for the construction of the genome-streamlined strains in the laboratory.

### Construction and characterization of the genome-streamlined strains

**Fig 3:**
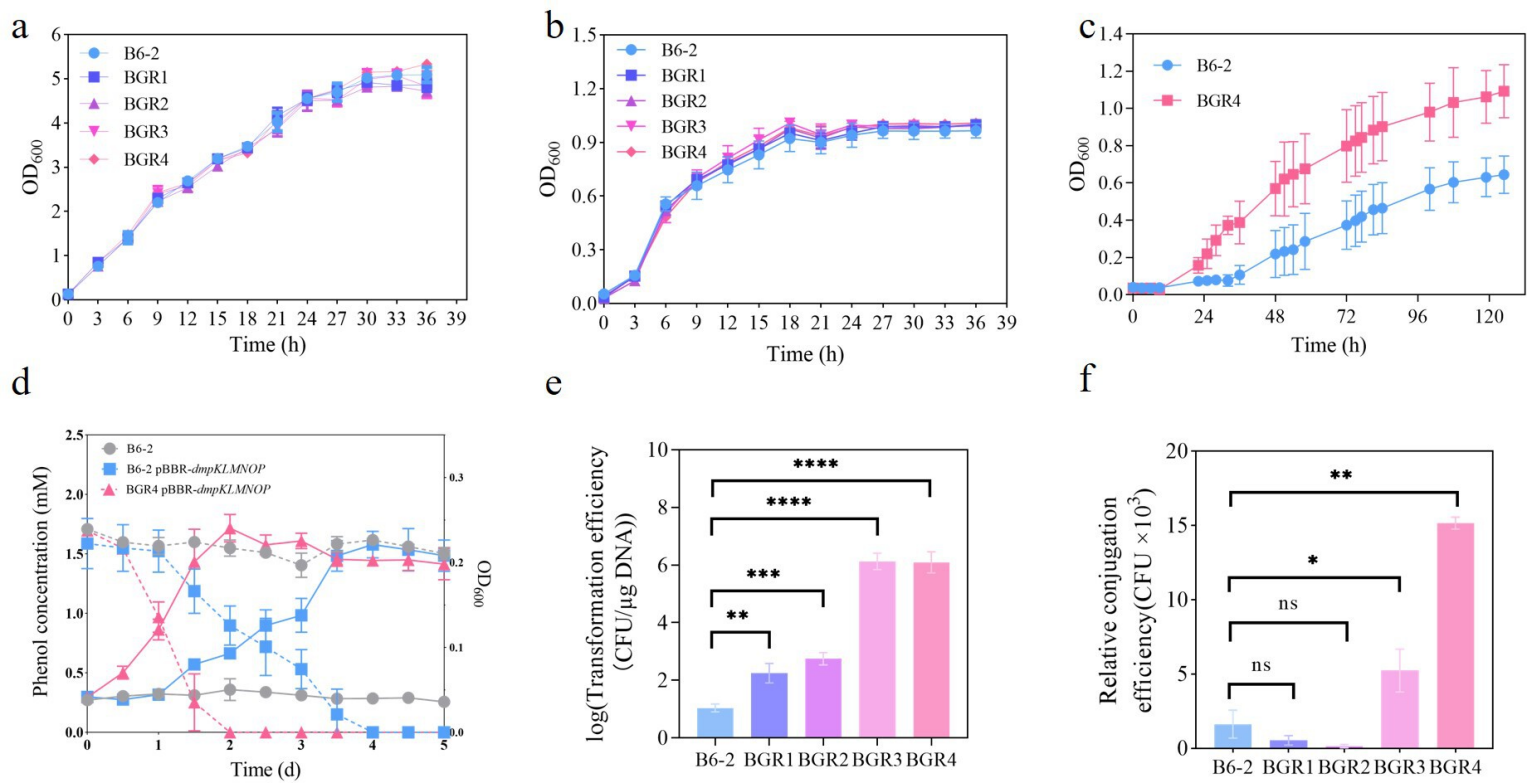
Gross physiological properties of *P. putida* B6-2 and its genome-streamlined strain(s). The growth curves of *P. putida* B6-2 and its genome-streamlined strain(s) in LB medium (**a**) and MSM supplemented with 2 g/L glucose (**b**) or 10 g/L glycerol (**c**). (**d**) The phenol degradation (shown as a dashed line) and growth (shown as a solid line) curves of *P. putida* B6-2, *P. putida* B6-2 pBBR-*dmpKLMNOP*, and *P. putida* BGR4 pBBR-*dmpKLMNOP*. (**e**) The electroporation efficiencies for the pBBR1MCS2 plasmid in *P. putida* B6-2 and its genome-streamlined strains. The vertical coordinate is the log value of the electroporation efficiency. CFU, colony-forming unit. (**f**) The conjugation efficiencies for the plasmid pK18mob*sacB*-Δ*vdh* into the *vdh* gene locus of *P. putida* B6-2 and its genome-streamlined strains. The data are presented as the means ± SDs from three independent experiments. One-way analysis of variance (ANOVA) was performed for statistical analysis. ns no significant difference, *P < 0.05, **P < 0.01, ***P < 0.001, and ****P < 0.0001.

The deletion targets were deleted using the traceless deletion method with the pK18mob*sacB* plasmid (Fig. S1). To verify the accurate deletion of the targeted regions, PCR and DNA sequencing of the amplified fragments were performed using the corresponding primers (Fig. S2). The growth characteristics of *P. putida* B6-2 and its genome-streamlined strains were subsequently evaluated. Growth was tested in both LB medium and mineral salt medium (MSM) supplemented with a carbon source that induced different metabolic pathways, i.e., glycerol for gluconeogenesis or glucose for glycolysis. Prior to this, the morphologies of *P. putida* B6-2 and *P. putida* BGR4 were examined by transmission electron microscopy (TEM), and no noticeable differences were observed (Fig. S3). The growth curves of *P. putida* B6-2 and its genome-streamlined strains in LB medium and MSM supplemented with 2 g/L glucose showed that the growth rates and growth tendencies of the five strains were similar, with no significant differences (Fig. 3a and b). Drop assays were also performed to evaluate the ability of the strains to grow on solid LB plates and solid mineral salt plates supplemented with 2 g/L glucose (Fig. S4a), and the results were consistent with the growth curve results mentioned above. However, the growth curves of *P. putida* B6-2 and *P. putida* BGR4 in glycerol were different, with *P. putida* BGR4 showing a 12-h reduction in the lag period, a higher growth rate, and a 0.7-fold increase in the maximum optical density at 600 nm (OD_600_) (Fig. 3c). Additionally, the ability of *P. putida* B6-2 and *P. putida* BGR4 to degrade phenol after heterologous expression of the phenol monooxygenase encoded by *dmpKLMNOP* from *Cupriavidus pinatubonensis* JMP134 (41) was evaluated. *P. putida* BGR4 pBBR-*dmpKLMNOP* exhibited a 24-h reduction in lag period, a higher growth rate, and a 1.1-fold increase in maximum phenol degradation rate compared with *P. putida* B6-2 pBBR-*dmpKLMNOP* (Fig. 3d). In conclusion, the findings above suggested that genome streamlining did not hinder the metabolic capacity of the genome-streamlined strains but actually enhanced the glycerol utilization ability of *P. putida* BGR4 and the phenol degradation capability of *P. putida* BGR4 pBBR-*dmpKLMNOP*.

The ability to accept and uptake exogenous DNAs is important for an ideal chassis cell. Therefore, the ability of *P. putida* B6-2 and its genome-streamlined strains to uptake foreign DNA by electroporation was tested using the broad host range vector pBBRMCS2. Compared with *P. putida* B6-2, the genome-streamlined strains exhibited varying degrees of improvement in electroporation efficiency, with *P. putida* BGR4 demonstrating the greatest increase, surpassing *P. putida* B6-2 by five orders of magnitude (Fig. 3e). The enhanced electroporation abilities of the genome-streamlined strains were mainly attributed to the knockout of two deoxyribonuclease genes, *endA-*1 and *endA-*2, while the knockout of the *hsdR* gene did not significantly affect electroporation efficiency (Fig. S5). In addition, bacteria can also obtain foreign DNA through conjugation. Gong et al. reported that the *vdh* gene locus was a favorable chromosomal integration site (6). Hence, the suicide plasmid pK18mob*sacB* was used to construct a chromosomal integration vector targeting the *vdh* gene locus. Compared with *P. putida* B6-2, both *P. putida* BGR3 and *P. putida* BGR4 exhibited significantly greater conjugation efficiencies, with *P. putida* BGR4 exhibiting the greatest increase (8.3-fold) (Fig. 3f). The deletion of the *endA*-1 and *endA*-2 genes, as well as the endonuclease-encoding gene of prophage 3, might explain the enhanced conjugation ability of *P. putida* BGR3 and *P. putida* BGR4. Taken together, the above results demonstrated that genome streamlining greatly enhanced the electroporation and conjugation efficiencies of *P. putida* BGR4.

### Transcriptomic analysis indicated more active metabolism within *P. putida* BGR4

**Fig 4:**
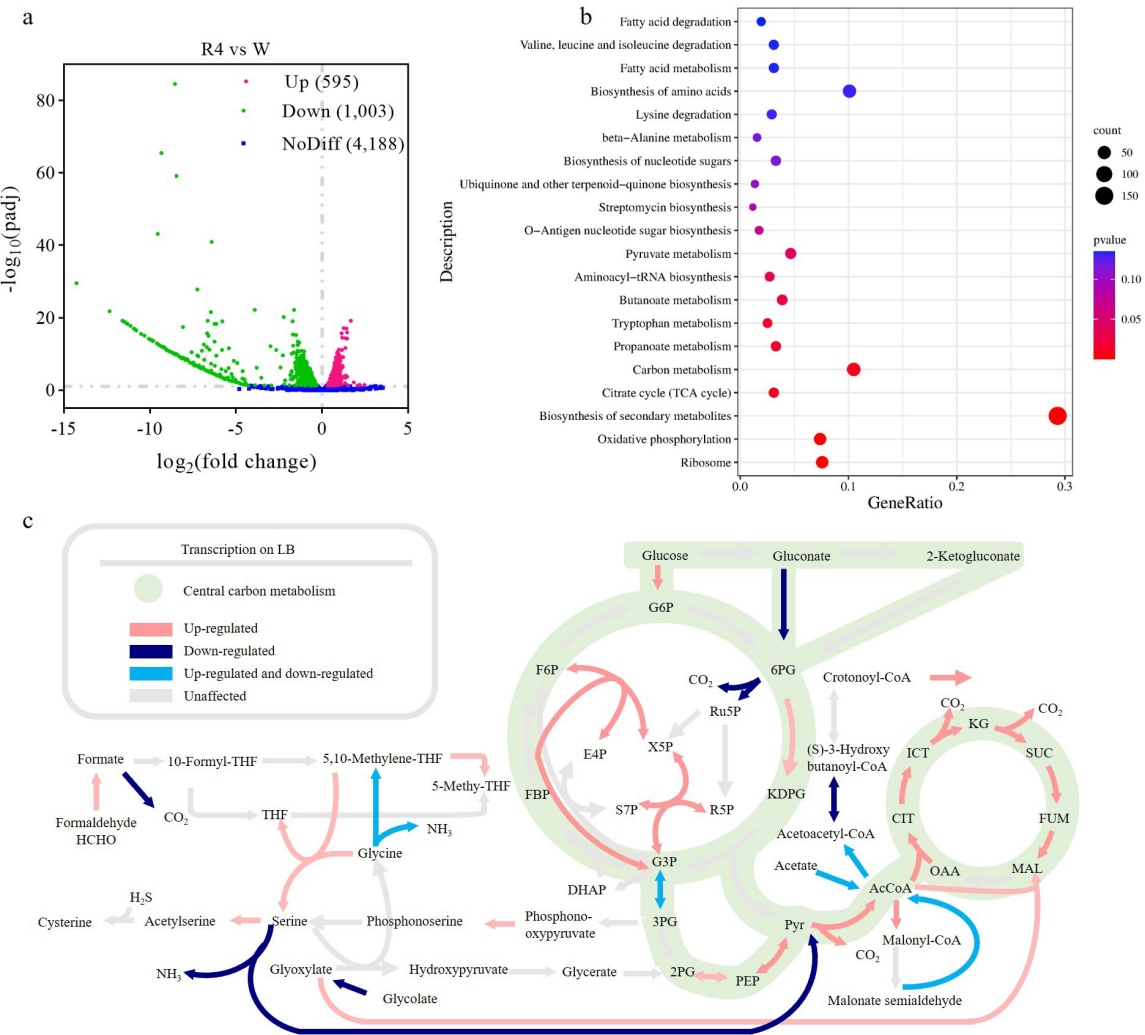
Transcriptomic analysis of DEGs between *P. putida* B6-2 and *P. putida* BGR4 in LB medium. Volcano map (**a**)and KEGG pathway enrichment analysis (**b**)of DEGs in *P. putida* BGR4 compared with *P. putida* B6-2. (**c**)DEGs enriched in the “carbon metabolism” pathway in *P. putida* BGR4 compared with *P. putida* B6-2. The pink arrows represent the exclusive upregulation of DEGs in the pathway. The dark-blue arrows represent the exclusive downregulation of DEGs in the pathway. The light-blue arrows indicate the presence of both upregulated and downregulated DEGs in the pathway. The gray arrows indicate that there was no significant difference. W, *P. putida* B6-2 cultured in LB medium; R4, *P. putida* BGR4 cultured in LB medium.

As mentioned previously, the GEMs predicted altered carbon metabolic capabilities between *P. putida* B6-2 and *P. putida* BGR4. Therefore, a transcriptomic analysis was subsequently performed to investigate how genome streamlining affects the basal physiological metabolism of *P. putida* BGR4. As the volcano plots showed, 595 differentially expressed genes (DEGs) were upregulated and 1,003 DEGs were downregulated in *P. putida* BGR4 compared with *P. putida* B6-2 (Fig. 4a). After KEGG pathway enrichment analysis, DEGs were found to be significantly enriched in the “biosynthesis of secondary metabolites,” “carbon metabolism,” “ribosome,” “oxidative phosphorylation,” and “tricarboxylic acid (TCA) cycle” pathways (Fig. 4b). Among these, the enrichment of DEGs in the “carbon metabolism” pathway indicated altered carbon metabolic capabilities between *P. putida* B6-2 and *P. putida* BGR4, which was consistent with the GEM-predicted results. Moreover, the expression of the majority of the DEGs involved in central carbon metabolism pathways (Fig. S6) was also significantly upregulated, indicating a greater metabolic capacity of *P. putida* BGR4 (Fig. 4c).

**Fig 5:**
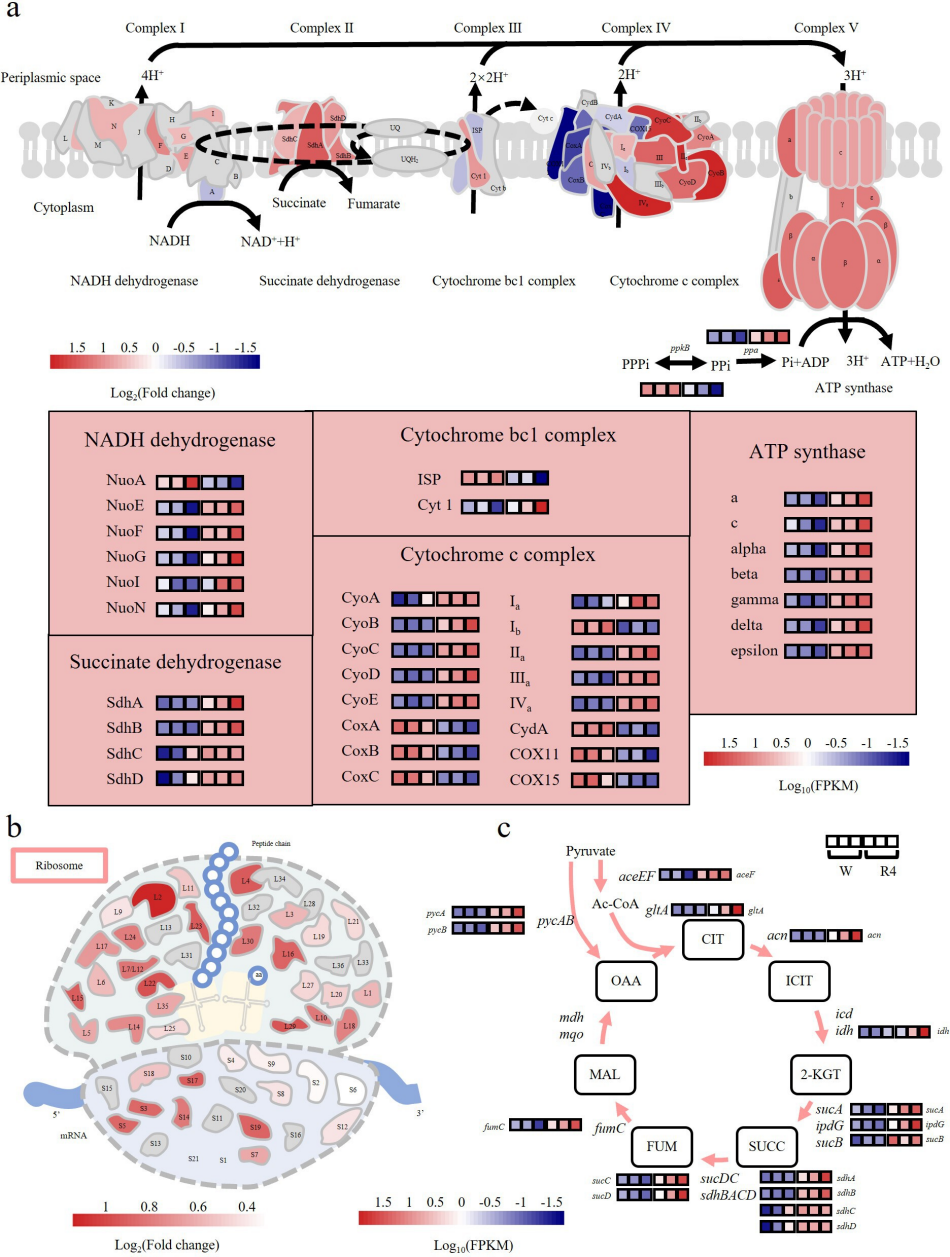
Analysis of the DEGs of *P. putida* BGR4 in LB medium compared with *P. putida* B6-2. (**a**) Fold changes and heatmap of transcripts in the “oxidative phosphorylation” pathway. (**b**) Fold changes in transcript expression in the “ribosome” pathway. (**c**) Heatmap of transcripts in the “TCA cycle” pathway. The components and structures of the “oxidative phosphorylation,” “TCA cycle,” and “ribosome” pathways are depicted based on the KEGG database. The gray blocks represent areas with no significant difference. W, *P. putida* B6-2 cultured in LB medium; R4, *P. putida* BGR4 cultured in LB medium.

The “oxidative phosphorylation” pathway is the primary pathway for generating the energy currency ATP to sustain basic life activities. Most of the DEGs involved in the “oxidative phosphorylation” pathway, such as the genes encoding some subunits of the complex of NAD(P)H dehydrogenase, cytochrome c oxidase, and succinic dehydrogenase, as well as seven of the eight subunits of ATP synthetase, were significantly upregulated (Fig. 5a). Moreover, *P. putida* BGR4 had higher ATP content, but there was no significant difference compared with that of *P. putida* B6-2 (Fig. S7). The TCA cycle is the main pathway for energy production, while ribosomes are responsible for protein synthesis. The overall DEGs in the “TCA cycle” and “ribosome” pathways were significantly upregulated, which also indicated more active metabolism within *P. putida* BGR4 (Fig. 5b and c).

### The genome-streamlined strains had a stronger ability to cope with stress

**Fig 6:**
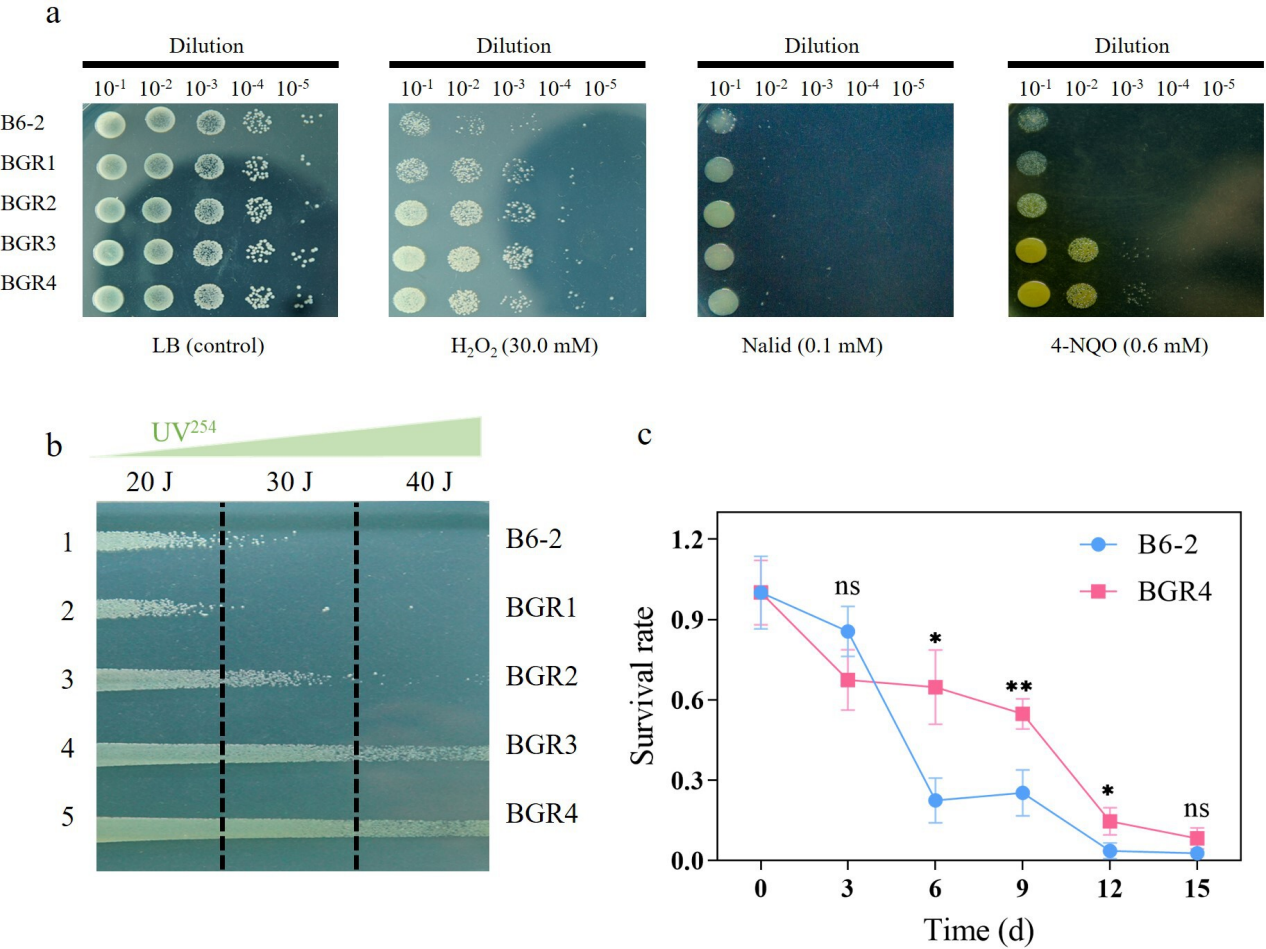
Survival of *P. putida* B6-2 and its genome-streamlined strain(s) under different stresses. (**a**) Drop assays to compare the fitness of *P. putida* B6-2 and its genome-streamlined strains under treatment with different chemical agents. (**b**) Plate tests to compare the tolerance of *P. putida* B6-2 and its genome-streamlined strains to UV irradiation. (**c**) Long-term survival tests comparing the ability of *P. putida* B6-2 and *P. putida* BGR4 to cope with starvation. The data are presented as the means ± SDs from six independent experiments. Two-tailed Student’s *t* tests were performed for statistical analysis. ns, no significant difference, *P < 0.01 and **P < 0.001.

The ability of chassis cells to withstand a stressful environment is the fundamental prerequisite for successful expression and functioning of contaminant degradation genes. Therefore, the tolerance of *P. putida* B6-2 and its genome-streamlined strains to acidic pH, alkaline pH, heat, osmotic pressure, oxidative stress, and DNA damage was measured using drop assays. The results showed that there was no significant difference in the tolerance to acidic pH, alkaline pH, heat, or osmotic pressure among *P. putida* B6-2 and its genome-streamlined strains (Fig. S4b through d). However, when exposed to oxidative stress and DNA damage induced by chemical agents (H_2_O_2_, nalidixic acid [Nalid] [42] or 4-nitroquinoline-1-oxide [4-NQO] [43, 44]), the genome-streamlined strains exhibited a greater survival rate than that of *P. putida* B6-2 (Fig. 6a), indicating that genome streamlining enhanced the tolerance of the genome-streamlined strains to oxidative stress and DNA damage.

In addition, UV irradiation and starvation are common adverse conditions encountered by chassis cells in the environment. Therefore, *P. putida* B6-2 and its genome-streamlined strains were streaked on the surface of LB plates and exposed to different intensities of UV radiation (254 nm). The results revealed that the genome-streamlined strains *P. putida* BGR2, *P. putida* BGR3, and *P. putida* BGR4 showed greater tolerance to UV irradiation (Fig. 6b). The starvation experiments were carried out by long-term cultivation without changing the medium. Compared with that of *P. putida* B6-2, the survival rate of *P. putida* BGR4 decreased significantly slower (Fig. 6c), showing that genome streamlining improved the starvation tolerance of *P. putida* BGR4.

### The presence of prophages reduced the resistance of *P. putida* B6-2 to 4-NQO

**Fig 7:**
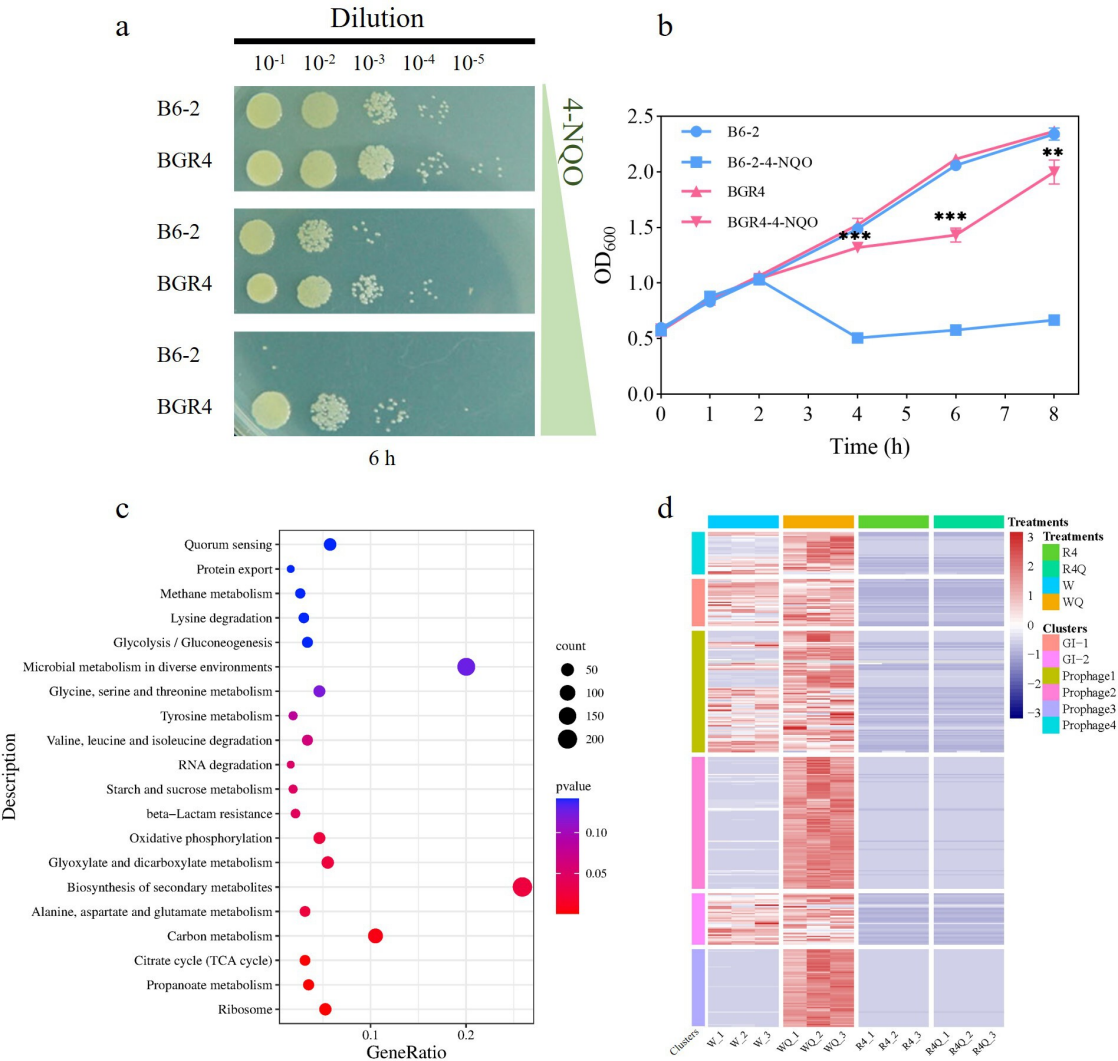
Cell survival and transcriptional analysis in the presence or absence of 4-NQO. (**a**) Drop assays of *P. putida* B6-2 and *P. putida* BGR4 grown in LB medium supplemented with 0.3 mM, 0.6 mM, or 1.2 mM 4-NQO for 6 h. (**b**) Growth curves of *P. putida* B6-2 and *P. putida* BGR4 in LB medium without or supplemented with 0.6 mM 4-NQO. (**c**) KEGG pathway enrichment analysis of DEGs in the treatment group. (**d**) Transcriptional analysis of the deleted regions between the control group and treatment group. W, *P. putida* B6-2 cultured in LB medium; R4, *P. putida* BGR4 cultured in LB medium; WQ, *P. putida* B6-2 cultured in LB medium supplemented with 0.6 mM 4-NQO; R4Q, *P. putida* BGR4 cultured in LB medium supplemented with 0.6 mM 4-NQO. The data are presented as the means ± SDs from three independent experiments. Two-tailed Student’s t tests were was performed for statistical analysis. ns, no significant difference, *P < 0.05, **P < 0.01, and ***P < 0.001.

As mentioned above, in the drop assays, the genome-streamlined strain *P. putida* BGR4 showed greater resistance to the typical reactive oxygen species (ROS) inducer and the potent mutagen 4-NQO, which was added to solid plates at a concentration of 0.6 mM. To explore how genome streamlining affected the tolerance of *P. putida* BGR4 to the ROS and DNA damage caused by 4-NQO, a transcriptomic analysis was performed. Prior to this, *P. putida* B6-2 and *P. putida* BGR4 were cultured in LB medium supplemented with different concentrations of 4-NQO to determine the appropriate 4-NQO treatment concentration and duration. By combining the results of the drop assays (Fig. 7a) and the growth curves (Fig. 7b), the 4-NQO treatment concentration and duration were selected as 0.6 mM and 4 h, respectively. Then, a transcriptomic analysis was performed.

First, the overall difference in gene expression between the control group (*P. putida* B6-2 and *P. putida* BGR4 cultured in LB medium) and the treatment group (*P. putida* B6-2 and *P. putida* BGR4 cultured in LB medium supplemented with 0.6 mM 4-NQO) was investigated. The results of cluster analysis showed that the transcripts in the same group were clearly correlated (Fig. S8), indicating that the experimental treatment and group setting were reasonable.

As shown in the volcano plots, 1,059 DEGs were upregulated and 1,377 DEGs were downregulated under 4-NQO treatment in *P. putida* BGR4 compared with *P. putida* B6-2 (Fig. S9). However, KEGG pathway enrichment analysis revealed that DEGs were not significantly enriched in the oxidative damage- or the DNA damage repair-related pathways (Fig. 7c). Moreover, the gene expression of prophage 2, prophage 3, and prophage 4 in *P. putida* B6-2 greatly increased upon treatment with 4-NQO (Fig. 7d).

**Fig 8:**
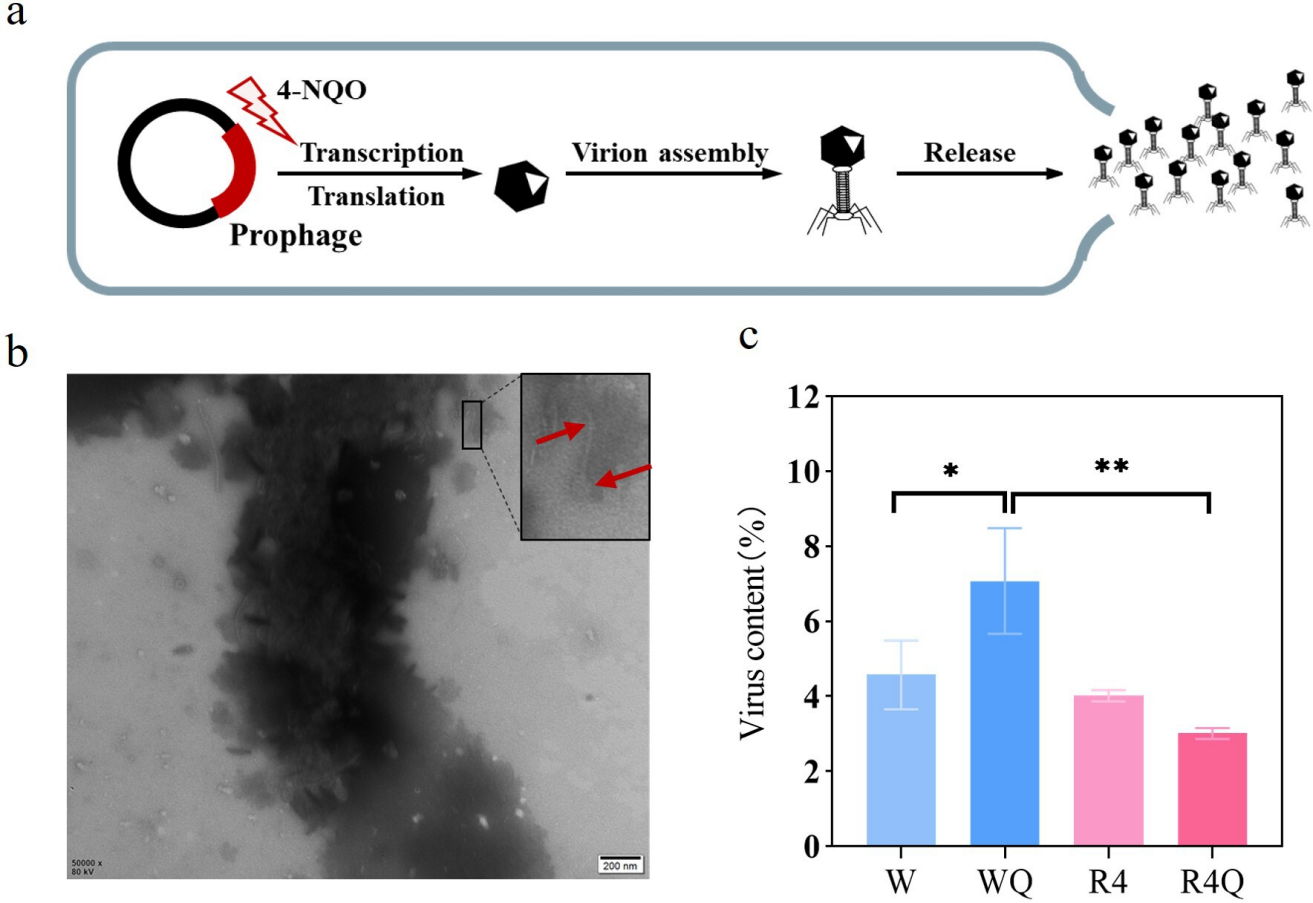
The induction of prophages and detection of virions. (**a**) The proposed schematic diagram of a prophage within *P. putida* B6-2 being induced by 4-NQO into the lytic cycle. (**b**) Morphology of the virion observed by TEM in the 4-NQO-treated bacterial culture of *P. putida* B6-2. (**c**) Flow cytometry analysis of the virion contents in the control group and treatment group. The data are presented as the means ± SDs from three independent experiments. W, *P. putida* B6-2 cultured in LB medium; R4, *P. putida* BGR4 cultured in LB medium; WQ, *P. putida* B6-2 cultured in LB medium supplemented with 0.6 mM 4-NQO; R4Q, *P. putida* BGR4 cultured in LB medium supplemented with 0.6 mM 4-NQO. One-way ANOVA was performed for statistical analysis. ns, no significant difference, *P < 0.05, **P < 0.01, and ***P < 0.001.

Prophages can interrupt the lysogenic state and enter the lytic cycle in response to chemical inducers, forming free virions that can lyse bacteria and cause bacterial death (45). Therefore, we hypothesized that 4-NQO might induce the prophages of *P. putida* B6-2 into the lytic cycle and cause bacterial cell death (Fig. 8a). A series of experiments were subsequently performed. First, virions were observed in the 4-NQO-treated bacterial culture of *P. putida* B6-2, demonstrating that 4-NQO could indeed induce a prophage-to-virion transition in *P. putida* B6-2 (Fig. 8b). Then, a phage infection test was carried out with mitomycin C (46) as the positive control. The results showed that the supernatant from *P. putida* B6-2 treated with 4-NQO did not form phage plaques on *P. putida* B6-2 lawns (data not shown), whereas the supernatant from mitomycin C treatment did (Fig. S10), indicating that 4-NQO-induced virions could not infect *P. putida* B6-2, unlike mitomycin C-induced virions. Flow cytometry was used to quantify the production of virions, and the supernatant produced by centrifugation after mitomycin C treatment of *P. putida* B6-2 was used as the positive control. The flow cytometry results showed that *P. putida* B6-2 produced more virions after 4-NQO treatment (Fig. 8c), which was consistent with the transcriptomic analysis in which the gene expression of the prophages of *P. putida* B6-2 greatly increased after 4-NQO treatment (Fig. 7d). In summary, the above experimental results indicated that the prophages within *P. putida* B6-2 could be induced by 4-NQO and transformed into the lytic cycle, leading to the formation of additional virions and causing bacterial death, thereby reducing the tolerance of *P. putida* B6-2 to 4-NQO.

## DISCUSSION

In recent years, genome streamlining has become an effective strategy for constructing ideal chassis cells. Various genome-streamlined chassis cells, such as those of *E. coli* (23), *Lactococcus lactis* (20), and *Streptomyces avermitilis* (47), have been successfully developed and utilized as platforms for product synthesis (48). However, little attention has been given to the construction of chassis cells that degrade contaminants, and Liang’s report (16) is the only study describing the construction of a chassis cell suitable for bioremediation by genome streamlining of *P. putida* KT2440. In this study, *P. putida* B6-2 was optimized by genome streamlining to further enhance its properties. Interestingly, both Liang’s report and our research indicated that genome streamlining improved the transformation efficiency, carbon source utilization capacity, and pollutant degradation capacity of the genome-streamlined strains, demonstrating the potential application of genome streamlining in constructing chassis strains suitable for bioremediation.

Although there have been numerous studies about target genome streamlining, these studies have been conducted in a somewhat blinded manner. Researchers could evaluate target traits only after laborious construction of genome-streamlined strains in the laboratory. However, genome streamlining does not always lead to improved phenotypes. Therefore, there remains a demand for a tool that can evaluate the effects of genome streamlining. In this study, GEMs were used to predict the effects of genome streamlining on the MGR, and the results of the GEM predictions showed that the genome-streamlined strain *P. putida* BGR4 had enhanced and the highest MGRs across all tested carbon resources (Fig. 2e). Therefore, strain BGR4 was chosen for *in vivo* construction. Subsequent growth experiments confirmed that *P. putida* BGR4 exhibited improved utilization of glycerol, as well as enhanced utilization of phenol after heterologous expression of the *dmpKLMNOP* gene cluster (Fig. 3c and d). Moreover, analysis of the FBA results of model wild_type_B6-2 and model BGR4 provided insights into why the genome-streamlined strain BGR4 has enhanced metabolic capabilities (Fig. 2c and d). In fact, GEMs have the potential to guide the construction of chassis cells not only for contaminant degradation purposes but also for other applications. For example, GEMs can be employed to guide the genome streamlining of chassis cells for biosynthetic purposes, with biomass serving as the objective function to solve for the maximum production rates. However, importantly, GEMs themselves have limitations. For example, the influence of global carbon regulation on carbon source utilization is not considered by GEMs (49). Within *P. putida* B6-2, prophage 1, prophage 3, and GI-1 contained several transcriptional regulators, prophage 3 specifically possessed a carbon storage regulator (Table S2). Both types of regulators can influence the carbon metabolic capacity of strains (50). The deletion of prophages and GIs in *P. putida* BGR4 might change its global carbon regulation system compared with that in *P. putida* B6-2. However, the GEMs did not account for the effect of those regulatory factors on carbon metabolism capability, which might lead to inconsistencies between the GEM-predicted results and the experimental results. For example, the GEMs predicted that *P. putida* BGR4 would also have enhanced glucose utilization ability, but the experimental results showed no significant difference in glucose utilization capacity between *P. putida* B6-2 and *P. putida* BGR4. In future studies, transcriptomic, proteomic, and metabolomic data can be incorporated into the GEMs to update and calibrate it, allowing the GEMs to more accurately reflect the actual metabolic state of the cell after genome streamlining (49). This, in turn, helps identify redundant genes and pathways and predicts the impact of further genome streamlining.

Many studies have reported that genome streamlining leads to many improved phenotypes, but few studies have reported the mechanisms underlying these improved phenotypes. In this study, we attempted to explain the reasons behind the superior phenotypes of the genome-streamlined strains by employing transcriptomic analysis. After genome streamlining, *P. putida* BGR4 exhibited enhanced utilization of not only glycerol but also phenol after heterologous expression of phenol monooxygenase. *P. putida* strains can convert glycerol to dihydroxyacetone phosphate (51), which then enters the glycolytic pathway and the TCA cycle (Fig. S11). *C. pinatubonensis* JMP134 can utilize phenol as the sole carbon source and convert phenol to catechol through the monooxygenase enzyme complex DmpKLMNOP (41). Catechol is then further catalyzed by a ring-cleaving enzyme to produce succinyl-CoA and acetyl-CoA, which ultimately enter the TCA cycle (Fig. S11). Transcriptomic analysis of *P. putida* B6-2 and *P. putida* BGR4 revealed that most of the DEGs involved in the downstream pathways of the EMP pathway and the TCA cycle were upregulated, while only one DEG involved in a reversible reaction was downregulated (Fig. 4c), which suggested that the upregulation of DEGs involved in the downstream pathway of EMP and the TCA cycle led to the enhanced utilization of glycerol by *P. putida* BGR4 and phenol by *P. putida* BGR4 pBBR-*dmpKLMNOP*. In fact, not only phenol but most aromatic compounds (such as benzene, naphthalene, and their analogs) are metabolized by bacteria via the catechol pathway and eventually enter the TCA cycle (4). Therefore, we considered *P. putida* BGR4 to be suitable for remediation of aromatic compound contamination.

Additionally, the genome-streamlined strains were more resistant to stress than *P. putida* B6-2. After the elimination of prophage 1, the genome-streamlined strains exhibited increased tolerance to H_2_O_2_ and Nalid (Fig. 6a). Under the stress of 4-NQO and UV irradiation, as the number of eliminated prophages increased, the resistance of the genome-streamlined strains to UV irradiation and 4-NQO gradually strengthened. After the elimination of the four prophages, *P. putida* BGR3 and *P. putida* BGR4 exhibited similar highest levels of 4-NQO and UV irradiation tolerance. Moreover, *P. putida* BGR3 and *P. putida* BGR4 consistently displayed similar highest stress tolerance abilities in all the stress tolerance tests. The above results suggested that the elimination of prophages enhanced the stress tolerance of the genome-streamlined strains, while the elimination of GIs might not. Transcriptomic analysis of the control group and treatment group revealed that the gene expression of prophage 2, prophage 3, and prophage 4 of *P. putida* B6-2 was strongly upregulated after 4-NQO treatment (Fig. 7d). Subsequent experiments confirmed that the prophages within *P. putida* B6-2 could indeed be induced by NQO, resulting in decreased tolerance of *P. putida* B6-2 to 4-NQO. In summary, the above results suggest it is the elimination of prophages that results mainly in improved stress resistance in *P. putida* BGR4, which is consistent with the findings of Esteban (52) and Qiao (20), who showed that the elimination of prophages increased the stress tolerance of genome-streamlined strains. Therefore, it is suggested that the stress tolerance of chassis cells may be improved by genome streamlining of prophages within chassis cells.

### Conclusions

Compared with the original strain *P. putida* B6-2, the genome-streamlined chassis strain *P. putida* BGR4 exhibited improvements in various physiological characteristics, such as electroporation and conjugation efficiencies, carbon source utilization, contaminant degradation capabilities, and stress tolerance. These desirable traits make *P. putida* BGR4 an optimum chassis strain for environmental remediation. Moreover, our study demonstrated the effectiveness of targeted genome streamlining based on bioinformatic analyses and GEM predictions in obtaining an optimized chassis cell for contaminant degradation. Additionally, the insights gained from the transcriptomic analysis, along with the established targeted genome streamlining procedure, can provide a valuable framework for guiding the genome streamlining of other strains for diverse applications.

## MATERIALS AND METHODS

### Bacterial strains and growth conditions

The bacterial strains and plasmids used in this work are listed in Table 1. The sequences of all the oligonucleotides used in this study are listed in Table S7. Bacteria were routinely grown in LB medium (10 g/L tryptone, 5 g/L yeast extract, and 10 g/L NaCl). LBS medium (10 g of tryptone, 5 g of yeast extract, 10 g of NaCl, and 150 g of sucrose per liter of distilled water, sterilized at 115°C for 15 min) was used for double-crossover integration screening. The MSM consisted of (g/L) 2.00 KH_2_PO_4_, 3.28 Na_2_HPO_4_·12H_2_O, 0.10 MgSO_4_, 1.00 (NH_4_)_2_SO_4_, and trace metals (0.050 CaCl_2_·2H_2_O, 0.050 CuCl_2_·2H_2_O, 0.008 MnSO_4_·H_2_O, 0.004 FeSO_4_·7H_2_O, 0.100 ZnSO_4_, 0.100 MoNa_2_O_4_·2H_2_O, and 0.050 K_2_WO_4_·2H_2_O). The solid media were prepared from liquid media supplemented with 1.5% (wt/vol) agar. If necessary, 2 g/L glucose, 10 g/L glycerol, or 2 mM phenol was added to the MSM as the sole carbon source. Phenol (≥99.5%, purity) was purchased from Sinopharm Chemical Reagent Co. Ltd. (Shanghai, China). Kanamycin (50 µg/mL) was added as needed. *P. putida* B6-2 and its genome-streamlined strains were cultured at 30°C, while *E. coli* cells were grown at 37°C. 2,6-Diaminopimelic acid (2,6-DAP) (0.3 mM) was required when cultivating *E. coli* WM3064. Other supplements were added to the media when needed as follows: 30 mM H_2_O_2_, 0.1 mM Nalid, and 0.6 mM 4-NQO. Cell growth was monitored by measuring the OD_600_.

**TABLE 1:** Detailed information on the bacterial plasmids and strains used in this study

Strain or plasmid	Description	Source
Strains		
*P. putida*		
B6-2	Wild-type strain	Professor Hongzhi Tang’s laboratory (12)
BGR1	B6-2 mutant (Δ*hsdR*, Δprophage 1)	This study
BGR2	B6-2 mutant (Δ*hsdR*, Δprophage 1, Δprophage 2)	This study
BGR3	B6-2 mutant (Δ*hsdR*, Δprophage 1, Δprophage 2, Δ*endA-*1, Δ*endA-*2, Δprophage 3, Δprophage 4)	This study
BGR4	B6-2 mutant (Δ*hsdR*, Δprophage 1, Δprophage 2, Δ*endA-*1, Δ*endA-*2, Δprophage 3, Δprophage 4, ΔGI-1, ΔGI-2)	This study
B6-2Δ*hsdR*	B6-2 mutant (Δ*hsdR*)	This study
BGR2Δ*endA-*1Δ*endA-*2	B6-2 mutant (Δ*hsdR*, Δprophage 1, Δprophage 2, Δ*endA-*1, Δ*endA-*2)	This study
B6-2 pBBR-*dmpKLMNOP*	B6-2 containing the plasmid pBBR-*dmpKLMNOP*	This study
BGR4 pBBR-*dmpKLMNOP*	BGR4 containing the plasmid pBBR-*dmpKLMNOP*	This study
*E. coli*		
DH5α	*supE*44 *lacU*169 (80*dlacZ*Δ*M*15) *hsdR*17	TransGen Biotech
WM3064	2,6-Diaminopimelic acid auxotroph	Laboratory stock
Plasmids		
pBBRMCS2	Empty vector for expression; Kan^r^	Laboratory stock
pK18mob*sacB*	Empty vector for allelic exchange; Kan^r^	Laboratory stock
pBBRMCS2-*dmpKLMNOP*	pBBRMCS2 derivative constructed for the expression of the phenol degradation gene cluster from *C. pinatubonensis* JMP134	Ren et al. (41)
pK18mob*sacB*-Δ*hsdR*	pK18mob*sacB* derivative containing PCR product covering up- and downstream regions of *hsdR*	This study
pK18mob*sacB*-Δprophage 1	pK18mob*sacB* derivative containing PCR product covering up- and downstream regions of prophage 1	This study
pK18mob*sacB*-Δprophage 2	pK18mob*sacB* derivative containing PCR product covering up- and downstream regions of prophage 2	This study
pK18mob*sacB*-Δ*endA-*1	pK18mob*sacB* derivative containing PCR product covering up- and downstream regions of *endA-*1	This study
pK18mob*sacB*-Δ*endA-*2	pK18mob*sacB* derivative containing PCR product covering up- and downstream regions of *endA-*2	This study
pK18mob*sacB*-Δprophage 3	pK18mob*sacB* derivative containing PCR product covering up- and downstream regions of prophage 3	This study
pK18mob*sacB*-Δprophage 4	pK18mob*sacB* derivative containing PCR product covering up- and downstream regions of prophage 4	This study
pK18mob*sacB*-ΔGI-1	pK18mob*sacB* derivative containing PCR product covering up- and downstream regions of GI-1	This study
pK18mob*sacB*-ΔGI-2	pK18mob*sacB* derivative containing PCR product covering up- and downstream regions of GI-2	This study
pK18mob*sacB*-Δ*vdh*	pK18mob*sacB* derivative containing PCR product covering up- and downstream regions of *vdh*	This study

### GEM construction, evaluation, and simulation

Gapseq (https://github.com/jotech/gapseq) was used for automatically reconstructing the GEMs. Gapseq uses a curated reaction database and a novel gap-filling algorithm. The resulting GEMs could be directly employed for FBA-based metabolic flux simulations of microbial growth. The GEMs in this study were constructed based on a recent genomic sequence of *P. putida* B6-2 (Refseq ID: CP015202.1) (53). First, the FASTA sequence of *P. putida* B6-2 was submitted to gapseq for semiautomated construction of the draft model; next, gap filling was conducted in the default LB medium. Subsequently, a literature comparison and model revision were conducted to ensure that the reactions and metabolic pathways in the model were consistent with known biological knowledge. Additionally, the parameters in the model were further calibrated: the upper and lower bounds of the reactions were adjusted to better reflect the state of the actual biological system; system constraints were defined with the upper bound for each metabolic reaction set to 1,000 and the lower bounds set to −1,000 and 0 for reversible and irreversible reactions, respectively, except for exchange reactions; the lower bounds of exchange reactions related to oxygen were set to −10, and the lower bounds of exchange reactions related to the medium compounds were set to −100, while the remaining bounds were set to 0. Models BGR1–4 were constructed by deleting or modifying genes, reactions, and metabolites of the model wild_type_B6-2. Moreover, the biomass objective function (BOF) for models BGR1-4 was derived by modifying the BOF of model wild_type_B6-2 based on the percentage of genome streamlining content. Specifically, the components in the BOF that included nucleotides or deoxyribonucleotides were proportionally adjusted. For example, in the BOF for the model wild_type_B6-2, the coefficient for ATP was 40.1654758653685, whereas in the BOF for BGR1, the coefficient for the ATP component was changed to 39.7638211067148 (40.1654758653685 * 0.99). All adjusted components included ATP, GTP, CTP, UTP, dATP, dGTP, dCTP, and dTTP. Detailed information and comparisons of metabolites and reactions among the five models are shown in Files 2 and S3. The SBML files of 5 GEMs could be accessed at https://github.com/fansiqing/models.git.

To ensure the reliability of the GEM-predicted results, the quality of the GEMs was evaluated using the model wild_type_B6-2 as an example. Memote was used to score the model wild_type_B6-2. The carbon source utilization capacity experiment was conducted as follows: 71 carbon sources from the GEN III Micro-Plate test and 7 reported positive carbon sources (12) were simulated in the model wild_type_B6-2. The predicted results were subsequently compared with the experimental results to evaluate the accuracy of the model wild_type_B6-2 in predicting the ability of *P. putida* B6-2 to utilize carbon sources. To ensure accurate simulation of the ability of *P. putida* B6-2 to utilize 78 carbon sources, additional transport reactions were added as free diffusion reactions within the model wild_type_B6-2 in MATLAB. Taking the addition of the phenol transport reaction as an example, the commands used were as follows: model = addReaction(model, 'EX_cpd00127_e0', 'cpd00127[c0] <=>'). The Memote reports for models BGR1-4 are presented in Files S5–S8. Models with scores above 70 in both assessments were considered relatively reliable. If the model scored below 70, further refinement of the model was recommended (27). In the quantitative assessment of the MGR on glucose, regression analysis was applied to calculate the specific growth rate of *P. putida* B6-2 on glucose, and the glucose maximum uptake rate of the model wild_type_B6-2 was referenced from *P. putida* KT2440 and defined as 6.1 mmol/g/h (54).

FBA was subsequently performed in MATLAB R2022b v.9.13 using the COBRA toolbox v.3.0 and the Gurobi linear solver to solve for the MGR. The commands used were as follows: model = changeObjective(wild_type_B6-2.xml, 'bio1'); solution = optimizeCbModel(model, 'max'). FVA was performed in the same environment as FBA. During FVA, the objective function value was fixed at the optimal value obtained through FBA under the condition of maximum growth rate, in order to analyze the upper and lower bounds of fluxes for each reaction. A reaction was defined as strictly constrained reaction if the relative change (relative change = |maxFlux − minFlux| / max(|maxFlux|, |minFlux|)) was less than a predefined threshold of 0.01. Here, minFlux represents the minimum flux, and maxFlux represents the maximum flux.

### Traceless deletion of redundant elements in *P. putida* B6-2

The plasmid pK18mob*sacB* (55) was used to construct the genome-streamlined strains (Fig. S1). First, homology arms of approximately 1,000 bp flanking the deleted regions were amplified and ligated with linearized pK18mob*sacB* vectors to construct knockout vectors. The vectors were subsequently transformed into *E. coli* WM3064. Conjugation of *E. coli* WM3064 with the *P. putida* strains resulted in single-crossover mutants. A single colony was picked into LB medium supplemented with 50 µg/mL kanamycin, followed by PCR to identify positive transformants. Next, the single-crossover mutant was cultured in LB medium without antibiotics at 30°C for 24 h, after which a small amount of the bacterial solution was coated on LBS solid medium. Then, a single colony was picked and cultured in LB medium with or without kanamycin. A single colony that could not grow in medium supplemented with kanamycin was chosen for analysis. PCR was used to confirm whether the single colony was a knockout mutant.

### Assessment of physiological characteristics

The electroporation efficiency was assessed by electroporation of the broad host range plasmid pBBRMCS2 (56) into *P. putida* B6-2 and the other four genome-streamlined strains at 1.4 kV for 2.5 ms, followed by CFU counting after 2 d of incubation at 30°C. The number of transformants was calculated as CFU per µg DNA. To measure conjugation efficiency, *P. putida* B6-2, the genome-streamlined strains and *E. coli* WM3064 pK18mob*sacB*-Δ*vdh* were grown in LB medium to OD_600_ = 0.6. Then, the bacterial cells were harvested (4,500 × *g* for 10 min) and washed twice with PBS (137.0 mM NaCl, 2.7 mM KCl, 10.0 mM Na_2_HPO_4_, and 2.0 mM KH_2_PO_4_; pH = 7.4). The donor and acceptor strains were mixed (6 mL:2 mL), and the mixture was resuspended in 50 µL of PBS. Then, 50 µL of the mixture was added to the center of an LB plate supplemented with 2,6-DAP and incubated for 12 h at 30°C. The bacteria were washed with 1 mL of PBS, and 10 µL of the bacterial mixture was collected and mixed with 90 µL of PBS. Then, 100 µL of the bacterial mixture was plated on an LB plate containing 50 µg/mL kanamycin and incubated for 1 d. Single colonies on the plate were counted to determine the conjugation efficiency. The Biolog GEN III Micro-Plate test was used to evaluate the metabolic phenotypes of *P. putida* B6-2. The culture steps were performed according to the manufacturer’s instructions. After the plates were incubated at 30°C for 48 h, the absorbance at 600 nm of the samples in the 96-well plate was measured to determine the metabolic phenotypes of *P. putida* B6-2. Well 1, where no carbon source was introduced, was designated the control. After subtraction of the control value, a positive OD_600_ value was taken as an indication of the ability to utilize the substrate. Conversely, a negative or zero OD_600_ value was considered to indicate a lack of ability to utilize the substrate.

### RNA extraction and RNA-seq analysis

Overnight cultures of *P. putida* B6-2 and *P. putida* BGR4 were inoculated into fresh LB medium with an initial OD_600_ = 0.05. When the OD_600_ of the cultures reached 0.6, the cultures in the treatment group were treated with 0.6 mM 4-NQO for 4 h, while the cultures in the control group were not treated. After completion of the treatment, samples for RNA extraction and RNA-seq were then immediately collected from the bacterial cultures. Three biological replicates of each sample were used to ensure data reliability. For RNA extraction, 1 mL of bacterial culture was collected at 13,000 × *g* for 2 min at 4°C, after which the medium was discarded, and the tubes were frozen in liquid nitrogen. The pellets were resuspended in 100 µL of lysozyme solution (20 mg/mL lysozyme in Tris-EDTA buffer, pH = 8.0). Total RNA was isolated using an Omega E.Z.N.A. Bacterial RNA Kit (Omega Bio-Tek, USA).

RNA sequencing was performed by Beijing Novogene Bioinformatics Technology Co. Ltd. (China). A total of 12 samples (four treatments with three replicates) were sequenced. The DEG analysis was performed using the DESeq R package (1.18.0). The resulting *P* values were subsequently adjusted using Benjamin and Hochberg’s approach to control for the false discovery rate. Genes with | log_2_(fold change) | > 0 and padj (adjusted *P*) < 0.05 were defined as DEGs. KOBAS software was used to test the statistical enrichment of DEGs in KEGG pathways. The figures were mainly generated with GraphPad Prism 8.01 (GraphPad Software Inc., San Diego, CA) or R using the heatmap package, and the parameters were modified when necessary.

### Determination of ATP content

The Beyotime ATP Test Kit S0026 was used to extract and determine the ATP content. The sample preparation and sampling time were in accordance with the RNA extraction method. The dosage of the bacterial solution was 0.1 OD_600_. The detection steps were carried out following the manufacturer’s instructions.

### Stress resistance

Acid stress and alkaline stress were achieved by adjusting the pH of the LB medium with HCl or NaOH, while osmotic stress was achieved by adding different concentrations of NaCl to the LB medium. Oxidative stress and DNA damage were induced by the chemical agents H_2_O_2_, Nalid, and 4-NQO. Nalid is a DNA-damaging antibiotic that can cause irreversible chromosome fragmentation, and 4-NQO is a potent mutagen that can induce transitions and transversions; both of these agents are ROS-producing agents and can cause DNA damage (42–44). When needed, 0.1 mM Nalid or 0.6 mM 4-NQO was added to the LB medium to induce oxidative stress and DNA damage. After the preparation of the above liquid media, 1.5% (wt/vol) agar was added, and the mixture was sterilized to prepare agar plates. Thermal stress was induced by incubating the plate at 38°C. For the above stress, overnight cultures of *P. putida* B6-2 and its genome-streamlined strains were diluted in PBS to an OD_600_ = 0.5, and 3 µL of each dilution was spotted onto agar plates. For oxidative stress and DNA damage induced by H_2_O_2_, activated cultures (OD_600_ = 0.05) of *P. putida* B6-2 and its genome-streamlined strains were treated with 30 mM H_2_O_2_ for 1 h and then diluted in PBS, after which 3 µL of each dilution was spotted onto LB agar plates. The UV irradiation tolerance test was performed as follows: 50 µL of activated cultures of *P. putida* B6-2 and its genome-streamlined strains (OD_600_ = 0.1) were spotted onto one side of the plate, after which the side containing 50 µL of culture was rotated 90° along the horizontal plane to form even lines. The plate was dried and exposed to UV light from a 254-nm lamp (CL-1000 Ultraviolet Crosslinker, 100 µJ/cm) at various intensities ranging from 20 J to 40 J, at a distance of 30 cm. Specifically, plates containing 4% (wt/vol) or 5% (wt/vol) NaCl were incubated at 30°C for 42 h and then photographed. Unless otherwise stated, the plates were photographed after 18 h of incubation at 30°C. In the starvation test, activated cultures of *P. putida* B6-2 and *P. putida* BGR4 were inoculated into fresh LB medium with an initial OD_600_ = 0.05 and subsequently cultured at 30°C for a total period of 15 d without the addition of fresh nutrients. CFU counts were determined by serial dilution of the cultures in PBS and plating 10 µL of each dilution on LB agar plates in six independent experiments.

### Flow cytometry

For the quantitative analysis of virions, sample preparation was performed with the RNA extraction method described above. After completion of the treatment, 1 mL of bacterial culture was centrifuged at 13,000 × *g* for 5 min, and the supernatant was collected for the quantitative analysis of virions. Moreover, overnight-grown cultures of *P. putida* B6-2 were treated with 2 µg/mL mitomycin C for 6 h, and the resulting supernatant was used as a positive control. Samples for flow cytometry analysis were prepared and detected according to the manufacturer’s instructions for the CytoFLEX flow cytometry instrument. Briefly, the supernatant was treated with pure and chilled ethanol to achieve a final ethanol concentration of 70% (vol/vol), and the mixture was then fixed overnight. SYBR dye (Invitrogen, 10,000×, DMSO dilution) was added to the mixture at a final concentration of 5×, after which the mixture was incubated for 10 min at 80°C in the dark. The excitation wavelength used to measure the fluorescence intensity of SYBR was 488 nm, and the emission wavelength was 509 nm. At least 100,000 events were counted for analysis. The data were analyzed with FlowJo 10.8.1.

### Phage infection test

The detailed procedures for the phage infection test were previously described by Esteban et al. (52). Briefly, overnight cultures of *P. putida* B6-2 were treated with 2 µg/mL mitomycin C for 6 h or with 0.6 mM 4-NQO for 4 h starting from an initial OD_600_ of 0.6. Then, 1 mL of culture was treated with chloroform, vortexed, and centrifuged at 13,000 × *g* for 5 min. Twenty-five microliters of the supernatant was spotted onto bacterial lawns prepared by mixing 100 µL of the overnight culture of *P. putida* B6-2 with 15 mL of LB medium containing 0.7% (wt/vol) agar and pouring the mixture onto an LB agar plate supplemented with 5 mM CaCl_2_ and 5 mM MgCl_2_. The control sample was LB medium supplemented with the same concentrations of mitomycin C and chloroform. The plate was then photographed after 18 h of incubation at 30°C.

### Imaging of bacteria and virions

A JEM-1400 Flash TEM (JEOL, Japan) was used to observe the morphology of *P. putida* B6-2, *P. putida* BGR4, and virions. Colonies of *P. putida* B6-2 and *P. putida* BGR4 on 0.5% (wt/vol) LB agar plates were dipped in 20 µL of ddH_2_O and resuspended to produce samples. To observe the morphology of virions induced by 4-NQO, the sample preparation procedure was the same as that for RNA extraction. Finally, 4 µL of each sample was transferred to a TEM carrier and stained with 1% (wt/vol) uranium acetate for detection.

### Phenol degradation assays

Ten milliliters of activated cultures of *P. putida* B6-2, *P. putida* B6-2 pBBR-*dmpKLMNOP*, and *P. putida* BGR4 pBBR-*dmpKLMNOP* in LB medium was harvested, washed twice with PBS, and then resuspended in MSM supplemented with 2 mM phenol. The strains were then passaged for an additional two generations in MSM supplemented with 2 mM phenol. Subsequently, the cells were harvested by centrifugation, washed twice with PBS, and resuspended in MSM supplemented with 2 mM phenol at an initial OD_600_ = 0.05. Cultures were incubated at 30°C and 200 rpm on a shaker. One milliliter of each culture was harvested at regular time intervals for the detection of the OD_600_ and phenol concentration. Analysis of the phenol concentration was performed by a GC-2014C gas chromatograph equipped with a flame ionization detector and an AOC-20i autoinjector (SHIMADZU, Shanghai, China). All the samples were centrifuged at 10,000 × *g* for 2 min. The supernatant was collected after pretreatment with a 0.45-µm filter. The detection program was as follows: the initial temperature was set at 60°C, the temperature was gradually increased to 160°C at a rate of 20°C/min, and the temperature was subsequently held at 160°C for 5 min.

## Data Availability

The transcriptome data have been deposited in the SRA database under the accession number PRJNA1058708.

## SUPPLEMENTAL MATERIAL

File S1 msystems.00845-24-s0001.docx Supplemental tables and figures.

File S2 msystems.00845-24-s0002.xlsx Reactions in models.

File S3 msystems.00845-24-s0003.xlsx Metabolites in models.

File S4 msystems.00845-24-s0004.html The Memote report of model wild_type_B6-2.

File S5 msystems.00845-24-s0005.html The Memote report of model BGR1.

File S6 msystems.00845-24-s0006.html The Memote report of model BGR2.

File S7 msystems.00845-24-s0007.html The Memote report of model BGR3.

File S8 msystems.00845-24-s0008.html The Memote report of model BGR4.

Legends msystems.00845-24-s0009.docx Supplemental material legends.

ASM does not own the copyrights to Supplemental Material that may be linked to, or accessed through, an article. The authors have granted ASM a non-exclusive, world-wide license to publish the Supplemental Material files. Please contact the corresponding author directly for reuse.
