# Supplementary material for: Genome streamlining of Pseudomonas putida B6-2 for bioremediation
Source: mSystems. 2024 Nov 12;9(12):e00845-24. doi: 10.1128/msystems.00845-24 (PMC11658094; doi:10.1128/msystems.00845-24)
Supplement: File S1 — Supplemental tables and figures. [file msystems.00845-24-s0001.docx]

**Additional File 1**

**Title:** Rational genome streamlining of *Pseudomonas putida* B6-2 for bioremediation

Siqing Fan^a, b^, Hao Ren^a, b^, Xueni Fu^a, b^, Xiangyu Kong^a, b^, Hao Wu^a, b^, Zhenmei Lu^a, b^

^a^ MOE Laboratory of Biosystem Homeostasis and Protection, College of Life Sciences, Zhejiang University, Hangzhou 310058, China

^b^ Cancer Center, Zhejiang University, Hangzhou 310058, China

Address to Siqing Fan, [12107037@zju.edu.cn](mailto:12107037@zju.edu.cn)

Address to Hao Ren, 0621735@zju.edu.cn

Address to Xueni Fu, 12207045@zju.edu.cn

Address to Xiangyu Kong, 0622263@zju.edu.cn

Address to Hao Wu, wuhaochs@hotmail.com

^#^Address correspondence to Zhenmei Lu, [lzhenmei@zju.edu.cn](mailto:lzhenmei@zju.edu.cn)

Table S1 Comparison of the degradation characteristics of *P. putida* KT2440 and *P. putida* B6-2.

| **Characteristic** | ***P. putida* KT2440** | ***P. putida* B6-2** |
| --- | --- | --- |
| **Degradation pathways of aromatic compounds** | The β-ketoadipate pathway (including p-coumarate, coniferyl alcohol, caffeate, quinate, 4-hydroxybenzoate and vanillate); the phenyl acetyl-coA pathway; the homogentisate pathway; the nicotinate pathway; the benzoate pathway | The β-ketoadipate pathway (including p-coumarate, coniferyl alcohol, caffeate, quinate, 4-hydroxybenzoate and vanillate); the phenyl acetyl-coA pathway; the homogentisate pathway; the nicotinate pathway; the benzoate pathway; the biphenyl pathway; the salicylate pathway |
| **Substrate spectrum of degradable contaminants** | Benzoate; catechol; 4-hydroxybenzoate | Benzoate; catechol; 4-hydroxybenzoate; salicylate; carbazole; dibenzothiophene; dibenzofuran; biphenyl; benzothiophene; dibenzo-p-dioxin; diphenyl ether;  4-bromodiphenyl ether;  4,4’-dichlorobiphenyl;  2,2’-dichlorobiphenyl;  2-chlorodibenzo-p-dioxin;  2,8-dichlorodibenzofuran;  3,4-dichlorobiphenyl;  4,4’-dibromodiphenyl ether;  4-methyldibenzothiophene;  4,6-dimethyldibenzothiophene;  2-methylbenzothiophene;  5-methylbenzothiophene;  fluorene; phenanthrene; anthracene; fluoranthene; pyrene; benzo[a]anthracene; chrysene; benzo[b]fluoranthene; benzo[k]fluoranthene; benzo[a]pyrene; dibenzo[a,h]anthracene;  benzo[g,h,i]perylene;  indeno[1,2,3-cd]pyrene |
| **Organic solvent resistant pumps** | (Ttg)ABC | (Ttg)ABC and (Srp)ABC |
| **Organic solvent tolerance** | Sensitive to 0.1% (v/v) toluene | Slight growth was detected when grown in LB medium supplemented with 20.0% (v/v) toluene; grew well in MSM with biphenyl under the stress of 0.5% (v/v) p-xylene; grew well in LB medium supplemented with 20.0% (v/v) n-decane, n-heptane, or p-xylene |

Table S2 Gene annotation of the deleted regions of *P. putida* B6-2.

| **Deletion unit and length** | **Position (start - end)** | **Proposed function** |
| --- | --- | --- |
| [***hsdR***](http://rast.nmpdr.org/seedviewer.cgi?page=Annotation&feature=fig\|6666666.290734.peg.6139) **(0.1 kb)** | 2,091,595-2,092,677 | type I restriction enzyme HsdR N-terminal domain-containing protein |
| ***endA-*1 (1.0 kb)** | 1,949,961-1,948,996 | endonuclease I precursor |
| ***endA-*2 (0.7 kb)** | 3,426,338-3,427,030 | endonuclease I precursor |
| **prophage 1 (66.3 kb)** | 1,233,431-1,232,367 | phage integrase |
|  | 1,233,671-1,233,435 | phage protein (ACLAME 1394) |
|  | 1,234,034-1,233,711 | hypothetical protein |
|  | 1,234,431-1,234,090 | hypothetical protein |
|  | 1,234,663-1,234,478 | hypothetical protein |
|  | 1,235,043-1,234,660 | hypothetical protein |
|  | 1,235,587-1,235,318 | hypothetical protein |
|  | 1,236,189-1,235,584 | hypothetical protein |
|  | 1,236,375-1,236,953 | protein of unknown function DUF88 |
|  | 1,237,107-1,237,850 | hypothetical protein |
|  | 1,237,980-1,238,159 | hypothetical protein |
|  | 1,238,271-1,238,369 | hypothetical protein |
|  | 1,238,459-1,238,728 | hypothetical protein |
|  | 1,239,510-1,238,818 | hypothetical protein |
|  | 1,239,623-1,240,324 | hypothetical protein |
|  | 1,241,245-1,240,430 | uncharacterized conserved protein |
|  | 1,241,619-1,241,272 | hypothetical protein |
|  | 1,242,166-1,241,708 | hypothetical protein |
|  | 1,242,432-1,242,163 | hypothetical protein |
|  | 1,242,835-1,242,452 | transcriptional regulator, LuxR family |
|  | 1,243,381-1,243,157 | FIG00962314: hypothetical protein |
|  | 1,244,081-1,244,404 | hypothetical protein |
|  | 1,244,488-1,246,871 | repeat region |
|  | 1,244,588-1,244,908 | mobile element protein |
|  | 1,244,905-1,245,240 | ISPpu14, transposase Orf2 |
|  | 1,245,304-1,246,839 | mobile element protein |
|  | 1,248,254-1,247,094 | hypothetical protein |
|  | 1,248,602-1,248,366 | hypothetical protein |
|  | 1,248,949-1,248,617 | hypothetical protein |
|  | 1,249,874-1,249,032 | phage repressor protein CI |
|  | 1,249,977-1,250,198 | hypothetical protein |
|  | 1,250,558-1,250,809 | hypothetical protein |
|  | 1,251,361-1,252,182 | phage DNA replication protein O |
|  | 1,252,179-1,253,576 | DNA helicase (EC 3.6.4.12), phage-associated |
|  | 1,253,576-1,253,782 | hypothetical protein |
|  | 1,253,764-1,254,063 | uncharacterized protein YbcO |
|  | 1,254,060-1,254,302 | hypothetical protein |
|  | 1,254,305-1,254,733 | hypothetical protein |
|  | 1,254,730-1,256,019 | phage integrase |
|  | 1,256,016-1,256,183 | hypothetical protein |
|  | 1,256,180-1,256,497 | hypothetical protein |
|  | 1,256,509-1,257,180 | hypothetical protein |
|  | 1,257,413-1,257,922 | lipid A 3-o-deacylase |
|  | 1,257,986-1,258,357 | methyl-accepting chemotaxis protein |
|  | 1,258,357-1,258,686 | lipoprotein, putative |
|  | 1,258,683-1,258,874 | hypothetical protein |
|  | 1,258,871-1,259,089 | hypothetical protein |
|  | 1,259,179-1,259,427 | FIG00954937: hypothetical protein |
|  | 1,259,586-1,260,086 | hypothetical protein |
|  | 1,260,090-1,261,769 | phage head, terminase DNA packaging protein A |
|  | 1,261,890-1,261,777 | hypothetical protein |
|  | 1,261,920-1,263,218 | phage portal protein |
|  | 1,263,235-1,264,092 | phage head, head-tail preconnector protease C |
|  | 1,264,095-1,265,276 | phage major capsid protein |
|  | 1,265,301-1,265,867 | hypothetical protein |
|  | 1,265,872-1,266,213 | phage protein, gp7 |
|  | 1,266,545-1,266,715 | FIG00957747: hypothetical protein |
|  | 1,266,719-1,267,294 | phage protein, HK97, gp10 |
|  | 1,267,287-1,267,673 | FIG00953725: hypothetical protein |
|  | 1,267,730-1,268,230 | hypothetical protein |
|  | 1,268,271-1,268,615 | hypothetical protein |
|  | 1,268,612-1,268,848 | hypothetical protein |
|  | 1,268,894-1,272,184 | hypothetical protein |
|  | 1,272,184-1,272,522 | phage minor tail protein |
|  | 1,272,519-1,273,268 | phage tail tip, assembly protein L |
|  | 1,273,271-1,274,026 | phage tail tip, assembly protein K |
|  | 1,274,023-1,274,616 | phage tail assembly protein I |
|  | 1,274,673-1,283,213 | phage tail tip, host specificity protein J |
|  | 1,283,210-1,283,590 | hypothetical protein |
|  | 1,283,574-1,283,744 | hypothetical protein |
|  | 1,283,807-1,284,025 | hypothetical protein |
|  | 1,284,491-1,284,132 | hypothetical protein |
|  | 1,284,755-1,284,546 | hypothetical protein |
|  | 1,284,856-1,285,395 | phage lysozyme R (EC 3.2.1.17) |
|  | 1,285,392-1,285,757 | hypothetical protein |
|  | 1,285,754-1,286,092 | phage protein (ACLAME 1146) |
|  | 1,286,236-1,286,400 | hypothetical protein |
|  | 1,286,981-1,288,048 | hypothetical protein |
|  | 1,288,190-1,288,414 | hypothetical protein |
|  | 1,288,488-1,288,781 | hypothetical protein |
|  | 1,288,778-1,289,083 | hypothetical protein |
|  | 1,291,329-1,292,642 | methyl-accepting chemotaxis sensor/transducer protein |
|  | 1,292,867-1,292,754 | hypothetical protein |
|  | 1,292,967-1,293,164 | hypothetical protein |
|  | 1,293,301-1,293,600 | hypothetical protein |
|  | 1,294,828-1,293,731 | NAD-dependent epimerase/dehydratase/hydrolase, alpha/beta fold family |
|  | 1,294,901-1,295,272 | transcriptional regulator, MerR family |
|  | 1,295,540-1,295,304 | hypothetical protein |
|  | 1,296,023-1,295,817 | hypothetical protein |
|  | 1,297,125-1,296,115 | alcohol dehydrogenase (EC 1.1.1.1) |
|  | 1,297,407-1,298,729 | repeat region |
|  | 1,298,040-1,297,456 | transposase |
|  | 1,298,107-1,298,253 | hypothetical protein |
|  | 1,298,675-1,298,277 | mobile element protein |
| **prophage 2 (59.6 kb)** | 1,751,965-1,750,781 | phage integrase |
|  | 1,752,311-1,752,135 | conserved hypothetical protein |
|  | 1,753,061-1,752,327 | Ren protein |
|  | 1,753,304-1,753,158 | hypothetical protein |
|  | 1,753,683-1,753,351 | conserved hypothetical protein |
|  | 1,754,102-1,753,671 | hypothetical protein |
|  | 1,754,584-1,754,099 | hypothetical protein |
|  | 1,754,838-1,754,584 | hypothetical protein |
|  | 1,755,188-1,754,835 | hypothetical protein |
|  | 1,755,547-1,755,185 | phage-associated homing endonuclease |
|  | 1,756,188-1,755,538 | phage protein |
|  | 1,756,980-1,756,273 | hypothetical protein |
|  | 1,757,041-1,757,298 | hypothetical protein |
|  | 1,759,118-1,757,301 | C-5 cytosine-specific DNA methylase family protein |
|  | 1,759,237-1,759,416 | hypothetical protein |
|  | 1,760,001-1,759,447 | hypothetical protein |
|  | 1,760,105-1,760,356 | hypothetical protein |
|  | 1,760,461-1,760,907 | hypothetical protein |
|  | 1,761,272-1,760,904 | hypothetical protein |
|  | 1,761,425-1,761,288 | hypothetical protein |
|  | 1,761,765-1,761,400 | hypothetical protein |
|  | 1,762,527-1,762,015 | hypothetical protein |
|  | 1,764,155-1,762,524 | hypothetical protein |
|  | 1,764,976-1,764,152 | phage recombination protein Bet |
|  | 1,765,143-1,765,259 | hypothetical protein |
|  | 1,765,467-1,765,264 | hypothetical protein |
|  | 1,765,634-1,765,464 | hypothetical protein |
|  | 1,765,858-1,765,631 | hypothetical protein |
|  | 1,766,283-1,765,855 | hypothetical protein |
|  | 1,766,546-1,766,304 | protein of unknown function DUF551 |
|  | 1,766,938-1,766,543 | hypothetical protein |
|  | 1,767,598-1,767,386 | hypothetical protein |
|  | 1,768,137-1,767,916 | hypothetical protein |
|  | 1,769,020-1,769,517 | hypothetical protein |
|  | 1,770,060-1,769,569 | hypothetical protein |
|  | 1,770,686-1,770,246 | hypothetical protein |
|  | 1,771,198-1,770,695 | hypothetical protein |
|  | 1,771,809-1,771,555 | hypothetical protein |
|  | 1,772,485-1,771,817 | phage CI repressor (ACLAME 5) |
|  | 1,772,594-1,772,770 | hypothetical protein |
|  | 1,772,801-1,773,007 | hypothetical protein |
|  | 1,773,027-1,773,227 | hypothetical protein |
|  | 1,773,568-1,773,332 | hypothetical protein |
|  | 1,773,729-1,774,586 | hypothetical protein |
|  | 1,774,583-1,775,038 | hypothetical protein |
|  | 1,775,035-1,775,814 | primosomal protein I |
|  | 1,775,811-1,776,572 | replication protein P |
|  | 1,776,575-1,776,955 | phage protein |
|  | 1,776,955-1,777,269 | hypothetical protein |
|  | 1,777,266-1,777,463 | hypothetical protein |
|  | 1,777,460-1,777,873 | hypothetical protein |
|  | 1,777,866-1,778,192 | hypothetical protein |
|  | 1,778,185-1,778,349 | hypothetical protein |
|  | 1,778,342-1,778,812 | hypothetical protein |
|  | 1,778,809-1,779,132 | hypothetical protein |
|  | 1,779,123-1,779,701 | phage recombination protein NinG |
|  | 1,779,698-1,779,844 | FIG00966827: hypothetical protein |
|  | 1,779,841-1,780,512 | FIG00954451: hypothetical protein |
|  | 1,780,907-1,780,983 | tRNA-Gly-TCC |
|  | 1,781,063-1,781,374 | FIG00964518: hypothetical protein |
|  | 1,781,374-1,781,643 | FIG00954293: hypothetical protein |
|  | 1,781,954-1,782,184 | NAD/FAD-utilizing enzyme, apparently involved in cell division |
|  | 1,782,195-1,782,359 | hypothetical protein |
|  | 1,782,356-1,782,790 | FIG00961435: hypothetical protein |
|  | 1,782,800-1,783,351 | hypothetical protein |
|  | 1,783,329-1,783,847 | FIG00947692: hypothetical protein |
|  | 1,783,961-1,784,578 | hypothetical protein |
|  | 1,784,650-1,786,041 | terminase large subunit |
|  | 1,786,041-1,787,417 | hypothetical protein |
|  | 1,787,421-1,788,464 | phage putative head morphogenesis protein, SPP1 gp7 family |
|  | 1,788,595-1,789,368 | hypothetical protein |
|  | 1,789,372-1,790,349 | phage capsid protein |
|  | 1,790,397-1,790,828 | hypothetical protein |
|  | 1,790,832-1,791,209 | FIG00961828: hypothetical protein |
|  | 1,791,212-1,791,595 | hypothetical protein |
|  | 1,791,595-1,791,975 | hypothetical protein |
|  | 1,791,972-1,792,394 | FIG00960076: hypothetical protein |
|  | 1,792,527-1,793,051 | phage Rha protein |
|  | 1,793,151-1,794,314 | hypothetical protein |
|  | 1,794,393-1,794,812 | hypothetical protein |
|  | 1,794,914-1,795,132 | hypothetical protein |
|  | 1,795,610-1,795,152 | hypothetical protein |
|  | 1,795,724-1,796,068 | hypothetical protein |
|  | 1,796,124-1,800,290 | hypothetical protein |
|  | 1,800,358-1,800,804 | hypothetical protein |
|  | 1,800,891-1,801,037 | hypothetical protein |
|  | 1,801,062-1,801,514 | hypothetical protein |
|  | 1,801,511-1,801,996 | domain of unknown function DUF1833 |
|  | 1,802,014-1,802,376 | hypothetical protein |
|  | 1,802,373-1,805,426 | hypothetical protein |
|  | 1,805,483-1,809,034 | hypothetical protein |
|  | 1,809,095-1,809,532 | structural protein P5, putative |
|  | 1,809,529-1,810,065 | hypothetical protein |
|  | 1,810,127-1,810,426 | hypothetical protein |
| **prophage 3 (37.7 kb)** | 4,198,615-4,198,166 | structural protein P5, putative |
|  | 4,201,350-4,198,675 | hypothetical protein |
|  | 4,202,225-4,201,380 | BNR domain protein |
|  | 4,202,765-4,202,310 | hypothetical protein |
|  | 4,205,777-4,202,775 | phage-related protein, tail component |
|  | 4,206,154-4,205,774 | Orf25 |
|  | 4,206,630-4,206,151 | hypothetical protein |
|  | 4,209,068-4,206,627 | phage tail, tail length tape-measure protein H |
|  | 4,209,655-4,209,500 | hypothetical protein |
|  | 4,210,194-4,209,724 | tail protein |
|  | 4,210,937-4,210,203 | major tail protein, putative |
|  | 4,211,371-4,211,003 | hypothetical protein |
|  | 4,211,862-4,211,368 | phage protein |
|  | 4,212,196-4,211,855 | head-tail adaptor |
|  | 4,212,672-4,212,196 | FIG00960774: hypothetical protein |
|  | 4,212,900-4,212,676 | hypothetical protein |
|  | 4,214,198-4,212,942 | phage major capsid protein |
|  | 4,214,909-4,214,208 | phage head maturation protease |
|  | 4,216,240-4,214,906 | phage portal protein |
|  | 4,216,397-4,216,233 | hypothetical protein |
|  | 4,218,118-4,216,409 | phage terminase, large subunit |
|  | 4,218,501-4,218,118 | phage terminase, small subunit |
|  | 4,219,019-4,218,645 | phage-associated homing endonuclease |
|  | 4,219,366-4,219,010 | hypothetical protein |
|  | 4,219,655-4,219,431 | probable acetyl/propionyl-CoA carboxylase alpha subunit (EC 6.3.4.14) |
|  | 4,220,003-4,219,848 | hypothetical protein |
|  | 4,220,387-4,220,055 | phage holin, lambda family |
|  | 4,221,152-4,220,643 | lipid A 3-o-deacylase |
|  | 4,221,767-4,221,378 | hypothetical protein |
|  | 4,222,051-4,221,764 | FIG00956875: hypothetical protein |
|  | 4,222,554-4,222,048 | FIG00959428: hypothetical protein |
|  | 4,223,947-4,222,541 | hypothetical protein |
|  | 4,224,723-4,223,944 | bacteriophage-encoded homolog of DNA replication protein DnaC |
|  | 4,225,514-4,224,720 | primosomal protein I |
|  | 4,225,741-4,225,511 | hypothetical protein |
|  | 4,226,301-4,225,738 | hypothetical protein |
|  | 4,226,612-4,226,298 | hypothetical protein |
|  | 4,227,382-4,226,609 | Roi protein |
|  | 4,227,675-4,227,379 | hypothetical protein |
|  | 4,227,917-4,227,672 | hypothetical protein |
|  | 4,228,219-4,227,914 | hypothetical protein |
|  | 4,228,470-4,228,216 | hypothetical protein |
|  | 4,228,916-4,228,614 | hypothetical protein |
|  | 4,229,030-4,229,770 | phage repressor protein CI |
|  | 4,229,907-4,230,299 | transcriptional regulator, LuxR family |
|  | 4,231,128-4,231,694 | phage protein |
|  | 4,231,694-4,232,173 | hypothetical protein |
|  | 4,232,259-4,232,681 | carbon storage regulator |
|  | 4,232,671-4,232,898 | alginate biosynthesis transcriptional activator |
|  | 4,233,059-4,232,937 | hypothetical protein |
|  | 4,233,092-4,233,781 | FIG00954594: hypothetical protein |
|  | 4,233,778-4,234,284 | hypothetical protein |
|  | 4,234,274-4,234,516 | FIG00954929: hypothetical protein |
|  | 4,234,753-4,235,943 | integrase |
| **prophage 4 (22.9 kb)** | 146,724*-*146,101 | FIG00965838: hypothetical protein |
|  | 147,048*-*146,803 | hypothetical protein |
|  | 147,614*-*149,062 | hypothetical protein |
|  | 150,138*-*150,356 | hypothetical protein |
|  | 150,360*-*150,536 | hypothetical protein |
|  | 150,529*-*150,819 | hypothetical protein |
|  | 151,137*-*151,295 | hypothetical protein |
|  | 151,292*-*151,555 | hypothetical protein |
|  | 151,552*-*151,884 | hypothetical protein |
|  | 151,881*-*152,186 | hypothetical protein |
|  | 152,183*-*152,398 | hypothetical protein |
|  | 152,395*-*153,111 | hypothetical protein |
|  | 153,114*-*153,398 | hypothetical protein |
|  | 153,391*-*153,618 | hypothetical protein |
|  | 153,615*-*154,505 | DNA primase, phage associated |
|  | 154,492*-*156,291 | DNA primase (EC 2.7.7.-), phage-associated |
|  | 156,628*-*156,930 | hypothetical protein |
|  | 156,927*-*157,526 | hypothetical protein |
|  | 157,530*-*158,474 | hypothetical protein |
|  | 158,651*-*159,262 | hypothetical protein |
|  | 159,538*-*159,750 | hypothetical protein |
|  | 159,747*-*162,650 | phage tail, tail length tape-measure protein H |
|  | 162,651*-*162,956 | hypothetical protein |
|  | 163,085*-*163,699 | resolvase |
|  | 164,359*-*164,628 | hypothetical protein |
|  | 164,792*-*165,343 | hypothetical protein |
|  | 165,354*-*166,250 | hypothetical protein |
|  | 166,576*-*166,818 | hypothetical protein |
|  | 167,580*-*167,783 | hypothetical protein |
|  | 168,124*-*168,435 | hypothetical protein |
|  | 168,451*-*168,699 | hypothetical protein |
|  | 169,028*-*168,822 | DNA-binding protein, putative |
| **GI-1 (29.1 kb)** | 693,506*-*692,808 | hypothetical protein |
|  | 693,964*-*693,752 | hypothetical protein |
|  | 694,085*-*693,957 | hypothetical protein |
|  | 698,202*-*695,059 | hypothetical protein |
|  | 698,407*-*698,682 | transposase InsO for insertion sequence element IS911 |
|  | 699,345*-*699,656 | transposase, IS3 family |
|  | 699,694*-*699,891 | mobile element protein |
|  | 702,025*-*700,067 | hypothetical protein |
|  | 702,900*-*702,022 | hypothetical protein |
|  | 703,827*-*702,901 | hypothetical protein |
|  | 704,897*-*703,827 | hypothetical protein |
|  | 706,183*-*704,909 | metallophosphoesterase |
|  | 706,931*-*706,482 | integrase |
|  | 707,109*-*707,276 | hypothetical protein |
|  | 707,459*-*707,346 | hypothetical protein |
|  | 709,245*-*708,091 | fic domain protein, YP3572 type |
|  | 709,568*-*709,496 | tRNA-Pseudo-CGT |
|  | 709,669*-*710,880 | phage integrase |
|  | 711,789*-*710,869 | hypothetical protein |
|  | 711,951*-*712,151 | excisionase, putative |
|  | 712,151*-*712,525 | hypothetical protein |
|  | 712,612*-*712,881 | hypothetical protein |
|  | 712,943*-*713,344 | hypothetical protein |
|  | 714,396*-*713,680 | hypothetical protein |
|  | 714,698*-*714,393 | hypothetical protein |
|  | 715,063*-*714,836 | hypothetical protein |
|  | 716,240*-*716,425 | hypothetical protein |
|  | 716,457*-*716,843 | hypothetical protein |
|  | 716,840*-*717,277 | hypothetical protein |
|  | 717,312*-*717,452 | hypothetical protein |
|  | 717,443*-*718,288 | hypothetical protein |
|  | 718,288*-*718,629 | hypothetical protein |
|  | 718,629*-*719,249 | phage tail tip, assembly protein I |
|  | 720,498*-*719,395 | hypothetical protein |
|  | 720,676*-*721,290 | resolvase |
|  | 721,896*-*721,291 | hypothetical protein |
| **GI-2 (41.5 kb)** | 2,465,239*-*2,464,973 | hypothetical protein |
|  | 2,465,393*-*2,465,869 | hypothetical protein |
|  | 2,466,388*-*2,466,501 | hypothetical protein |
|  | 2,466,560*-*2,467,819 | hypothetical protein |
|  | 2,467,803*-*2,469,590 | sulfur carrier protein adenylyltransferase ThiF |
|  | 2,469,772*-*2,469,638 | hypothetical protein |
|  | 2,470,621*-*2,470,070 | exonuclease, RNase T and DNA polymerase III |
|  | 2,470,930*-*2,470,727 | hypothetical protein |
|  | 2,471,536*-*2,471,372 | hypothetical protein |
|  | 2,471,666*-*2,472,784 | hypothetical protein |
|  | 2,472,875*-*2,475,438 | repeat region |
|  | 2,473,830*-*2,473,030 | mobile element protein |
|  | 2,475,331*-*2,473,823 | transposase |
|  | 2,475,523*-*2,475,392 | hypothetical protein |
|  | 2,476,318*-*2,475,566 | hypothetical protein |
|  | 2,477,754*-*2,476,315 | dihydropyrimidinase (EC 3.5.2.2) |
|  | 2,478,647*-*2,477,886 | oxidoreductase, short-chain dehydrogenase/reductase family |
|  | 2,479,615*-*2,478,644 | uricase (urate oxidase) (EC 1.7.3.3) |
|  | 2,480,549*-*2,479,608 | NAD-dependent epimerase/dehydratase |
|  | 2,482,204*-*2,480,546 | oligopeptide ABC transporter, ATP-binding protein OppD (TC 3.A.1.5.1) |
|  | 2,483,046*-*2,482,204 | ABC transporter, permease protein 2 |
|  | 2,484,083*-*2,483,043 | dipeptide ABC transporter, permease protein DppB (TC 3.A.1.5.2) |
|  | 2,485,762*-*2,484,152 | ABC transporter, substrate-binding protein |
|  | 2,486,179*-*2,486,910 | transcriptional regulator, GntR family |
|  | 2,488,043*-*2,487,150 | 2-hydroxy-3-oxopropionate reductase (EC 1.1.1.60) |
|  | 2,488,946*-*2,488,164 | hydroxypyruvate isomerase (EC 5.3.1.22) |
|  | 2,490,739*-*2,488,958 | glyoxylate carboligase (EC 4.1.1.47) |
|  | 2,490,847*-*2,491,749 | transcriptional regulator, LysR family, in glycolate utilization operon |
|  | 2,491,863*-*2,492,708 | hypothetical protein |
|  | 2,493,738*-*2,492,830 | cointegrate resolution protein S |
|  | 2,493,912*-*2,494,889 | Tn4651, cointegrate resolution protein T |
|  | 2,495,225*-*2,495,578 | hypothetical gene |
|  | 2,495,946*-*2,495,788 | hypothetical protein |
|  | 2,496,382*-*2,496,083 | hypothetical protein |
|  | 2,498,430*-*2,497,504 | hypothetical protein |
|  | 2,498,988*-*2,499,656 | hypothetical protein |
|  | 2,499,634*-*2,501,553 | hypothetical protein |
|  | 2,501,537*-*2,502,538 | GTP-binding protein, putative |
|  | 2,502,531*-*2,505,050 | hypothetical protein |
|  | 2,506,513*-*2,506,274 | RelB/StbD replicon stabilization protein (antitoxin to RelE/StbE) |

Table S3 Detailed information about the GEMs.

| **Model** | **Reaction** | **Metabolite** | **Gene** |
| --- | --- | --- | --- |
| **wild_type_B6-2.xml** | 2818 | 2363 | 1299 |
| **BGR1.xml** | 2818 | 2363 | 1299 |
| **BGR2.xml** | 2818 | 2363 | 1299 |
| **BGR3.xml** | 2818 | 2363 | 1299 |
| **BGR4.xml** | 2815 | 2360 | 1292 |

Table S4 Results of the GEN III Micro-Plate™ test and the wild_type_B6-2 model prediction.

| **Substrate** | **OD_600_** | **MGR (h^-1^)** | **Result** | |
| --- | --- | --- | --- | --- |
| **α-D-glucose** | 0.192 | 1.144207 | True positive |  |
| **Glycerol** | 0.140 | 0.660451 | True positive |  |
| **L-alanine** | 0.384 | 0.523820 | True positive |  |
| **L-arginine** | 0.176 | 0.890261 | True positive |  |
| **L-histidine** | 0.385 | 0.852036 | True positive |  |
| **Acetic acid** | 0.157 | 0.317467 | True positive |  |
| **D-galacturonic acid** | 0.202 | 0.797010 | True positive |  |
| **L-lactic acid** | 0.275 | 0.523820 | True positive |  |
| **L-aspartic acid** | 0.232 | 0.545828 | True positive |  |
| **Citric acid** | 0.170 | 0.801761 | True positive |  |
| **L-glutamic acid** | 0.303 | 0.805309 | True positive |  |
| **Mucic acid** | 0.185 | 0.812499 | True positive |  |
| **Propionic acid** | 0.348 | 0.499440 | True positive |  |
| **Quinic acid** | 0.261 | 0.455371 | True positive |  |
| **L-malic acid** | 0.238 | 0.537681 | True positive |  |
| **D-serine** | 0.218 | 0.440796 | True positive |  |
| **L-serine** | 0.208 | 0.440796 | True positive |  |
| **D-saccharic acid** | 0.211 | 0.805309 | True positive |  |
| **γ-Aminobutyric Acid** | 0.619 | 0.687659 | True positive |  |
| **L-pyroglutamic acid** | 0.354 | 0.744416 | True positive |  |
| **D-gluconic acid** | 0.634 | 0.997229 | True positive |  |
| **α-Keto-glutaric acid** | 0.080 | 0.716252 | True positive |  |
| **β-Hydroxy-D,L-butyric acid** | 0.225 | / | False negative |  |
| **D-glucuronic acid** | 0.202 | / | False negative |  |
| **D-mannose** | 0.102 | / | False negative |  |
| **L-galactonic acid lactone** | 0.124 | / | False negative |  |
| **Formic acid** | - | 0.074484 | False positive |  |
| **Dextrin** | - | 3.993458 | False positive |  |
| **D-maltose** | - | 1.803497 | False positive |  |
| **D-trehalose** | - | 1.803497 | False positive |  |
| **D-cellobiose** | - | 1.803497 | False positive |  |
| **Sucrose** | - | 1.803497 | False positive |  |
| **β-Methyl-D-glucoside** | - | 1.332182 | False positive |  |
| **D-fructose** | - | 1.158749 | False positive |  |
| **L-fucose** | - | 0.858408 | False positive |  |
| **Inosine** | - | 1.121181 | False positive |  |
| **D-sorbitol** | - | 1.232309 | False positive |  |
| **D-mannitol** | - | 0.068060 | False positive |  |
| **D-arabitol** | - | 1.049573 | False positive |  |
| **D-glucose-6-PO_4_** | - | 1.200295 | False positive |  |
| **α-Keto-butyric acid** | - | 0.666921 | False positive |  |
| **Acetoacetic acid** | - | 0.656429 | False positive |  |
| **D-fructose-6-PO_4_** | - | 1.200295 | False positive |  |
| **Methyl pyruvate** | - | / | True negative |  |
| **Bromo-succinic acid** | - | / | True negative |  |
| **Gentiobiose** | - | / | True negative |  |
| **Stachyose** | - | / | True negative |  |
| **D-raffinose** | - | / | True negative |  |
| **α-D-lactose** | - | / | True negative |  |
| **D-melibiose** | - | / | True negative |  |
| **N-acetyl-D-glucosamine** | - | / | True negative |  |
| **N-acetyl-β-D-mannosamine** | - | / | True negative |  |
| **N-acetyl-neuraminic Acid** | - | / | True negative |  |
| **D-salicin** | - | / | True negative |  |
| **D-turanose** | - | / | True negative |  |
| **D-galactose** | - | / | True negative |  |
| **3-Methyl glucose** | - | / | True negative |  |
| **L-rhamnose** | - | / | True negative |  |
| **myo-Inositol** | - | / | True negative |  |
| **Gelatin** | - | / | True negative |  |
| **Glycyl-L-proline** | - | / | True negative |  |
| **Pectin** | - | / | True negative |  |
| **Glucuronamide** | - | / | True negative |  |
| **p-Hydroxy-phenylacetic acid** | - | / | True negative |  |
| **D-lactic acid methyl ester** | - | / | True negative |  |
| **D-malic acid** | - | / | True negative |  |
| **Tween 40** | - | / | True negative |  |
| **α-Hydroxy-butyric acid** | - | / | True negative |  |
| **D-aspartic acid** | - | / | True negative |  |
| **N-acetyl-D-galactosamine** | - | / | True negative |  |
| **D-fucose** | - | / | True negative |  |

Note: The symbol '-' indicates no growth in the GEN III Micro-Plate™ test, while the symbol '/' indicates no growth in the wild_type_B6-2 model-predicted results. The maximum uptake rate of the substrates in the wild_type_B6-2 model was defined as 10 mmol/g/h.

Table S5 The utilization capabilities of *P. putida* B6-2 for 7 carbon sources predicted by the wild_type_B6-2 model.

| **Substrate** | **MGR (h^-1^)** | **Result** |
| --- | --- | --- |
| **Benzoate** | 0.291784 | True positive |
| **Salicylate** | 0.280645 | True positive |
| **Protocatechuate** | 0.420340 | True positive |
| **Biphenyl** | 0.283578 | True positive |
| **Catechol** | 0.419134 | True positive |
| **4-Hydroxybenzoate** | 0.274141 | True positive |
| **Carbazole** | / | False negative |

Note: The symbol '/' indicates no growth in the wild_type_B6-2 model-predicted results. The maximum uptake rate of the substrates in the wild_type_B6-2 model was defined as 10 mmol/g/h.

Table S6 GEM-predicted results for the MGRs (h^-1^) on LB, 28 positive carbon sources and phenol.

| **Substrate** | **wild_type_B6-2** | **BGR1** | **BGR2** | **BGR3** | **BGR4** |
| --- | --- | --- | --- | --- | --- |
| **LB** | 1.093899 | 1.1057058 | 1.1177706 | 1.1301017 | 1.1426335 |
| **α-D-glucose** | 1.144207 | 1.147685 | 1.151183 | 1.154703 | 1.158245 |
| **Glycerol** | 0.660451 | 0.662444 | 0.664448 | 0.666465 | 0.668494 |
| **L-alanine** | 0.523820 | 0.525413 | 0.527015 | 0.528628 | 0.530250 |
| **L-arginine** | 0.890261 | 0.897272 | 0.904395 | 0.911632 | 0.918985 |
| **L-histidine** | 0.852036 | 0.857481 | 0.862997 | 0.868584 | 0.874243 |
| **Acetic acid** | 0.317467 | 0.318432 | 0.319403 | 0.320380 | 0.321364 |
| **D-galacturonic acid** | 0.797010 | 0.802055 | 0.807164 | 0.812338 | 0.817580 |
| **L-lactate** | 0.523820 | 0.525413 | 0.527015 | 0.528628 | 0.530250 |
| **L-aspartic acid** | 0.545828 | 0.547490 | 0.549163 | 0.550846 | 0.552540 |
| **Citric acid** | 0.801761 | 0.804102 | 0.806457 | 0.808826 | 0.811208 |
| **L-glutamic acid** | 0.805309 | 0.807614 | 0.809932 | 0.812263 | 0.814607 |
| **Mucic acid** | 0.812499 | 0.814825 | 0.817163 | 0.819515 | 0.821881 |
| **Propionic acid** | 0.499440 | 0.502838 | 0.506284 | 0.509777 | 0.513318 |
| **Quinic acid** | 0.455371 | 0.458374 | 0.461416 | 0.464499 | 0.467624 |
| **L-malic acid** | 0.537681 | 0.539319 | 0.540967 | 0.542625 | 0.544293 |
| **D-serine** | 0.440796 | 0.442130 | 0.443473 | 0.444823 | 0.446182 |
| **L-serine** | 0.440796 | 0.442130 | 0.443473 | 0.444823 | 0.446182 |
| **D-saccharic acid** | 0.805309 | 0.807614 | 0.809932 | 0.812263 | 0.814607 |
| **α-Keto-glutaric acid** | 0.716252 | 0.718345 | 0.720449 | 0.722566 | 0.724695 |
| **γ-Aminobutyric acid** | 0.687659 | 0.693151 | 0.698732 | 0.704403 | 0.710167 |
| **L-pyroglutamic acid** | 0.744416 | 0.749634 | 0.754925 | 0.760292 | 0.765735 |
| **D-gluconic acid** | 0.997229 | 1.004554 | 1.011987 | 1.019531 | 1.027189 |
| **Benzoate** | 0.291784 | 0.293310 | 0.294852 | 0.296411 | 0.297986 |
| **Salicylate** | 0.280645 | 0.282089 | 0.283549 | 0.285023 | 0.286514 |
| **Protocatechuate** | 0.420340 | 0.422952 | 0.425596 | 0.428274 | 0.430986 |
| **Biphenyl** | 0.283578 | 0.285044 | 0.286525 | 0.288021 | 0.289534 |
| **Catechol** | 0.419134 | 0.421729 | 0.424355 | 0.427015 | 0.429708 |
| **4-Hydroxybenzoate** | 0.274141 | 0.275541 | 0.276957 | 0.278386 | 0.279831 |
| **Phenol** | 0.275069 | 0.276473 | 0.277892 | 0.279325 | 0.280773 |

Note: The maximum uptake rate of the substrates of LB in the GEMs was defined as 1 mmol/g/h, and that of other substrates was defined as 10 mmol/g/h.

Table S7 Sequences of primers used in this study.

| **Primer** | **Sequence (5’ > 3’)** | **Description** |
| --- | --- | --- |
| ***hsdR*-UP-F** | acatgattacgaattcacgcagtgcgtgagttcttc | Upstream flanking fragments |
| ***hsdR*-UP-R** | ataaggcaagcacaagtctcgctcctcgctcagat | Upstream flanking fragments |
| ***hsdR*-DOWN-F** | agcgaggagcgagacttgtgcttgccttatccgtg | Downstream flanking fragments |
| ***hsdR*-DOWN-R** | ggccagtgccaagcttaatgctgccatccggaatgc | Downstream flanking fragments |
| **V-B6-2-*hsdR*-UP-F** | acagcctggacaagatcatc | Δ*hsdR* strain verification |
| **V-*hsdR*-UP-F** | cggtggctgtgctactagga | Δ*hsdR* strain verification |
| **V-*hsdR*-DOWN-R** | acgccaccttaaaccgtgtc | Δ*hsdR* strain verification |
| **V-B6-2-*hsdR*-DOWN-R** | gccccacgtaaaatagagtc | Δ*hsdR* strain verification |
| **Prophage1-UP-F** | acatgattacgaattcagcctgcgcaacgaaatcac | Upstream flanking fragments |
| **Prophage1-UP-R** | tttccggagagtctgatcgctgatgcggaaacgat | Upstream flanking fragments |
| **Prophage1-DOWN-F** | ttccgcatcagcgatcagactctccggaaattcgg | Downstream flanking fragments |
| **Prophage1-DOWN-R** | ggccagtgccaagcttttctgcttagaggcgagctg | Downstream flanking fragments |
| **V-B6-2-Pro1-UP-F** | agcagaagaagggtaaggcc | Δprophage 1 strain verification |
| **V-Pro1-UP-F** | tgctccggattgcaaatccg | Δprophage 1 strain verification |
| **V-Pro1-DOWN-R** | tcaagcaggtgatcgtgctc | Δprophage 1 strain verification |
| **V-B6-2-Pro1-DOWN-R** | tggcgtaacgacagcaaggt | Δprophage 1 strain verification |
| **Prophage2-UP-F** | acatgattacgaattcaggttgtcgagcgatacagg | Upstream flanking fragments |
| **Prophage2-UP-R** | ccttcctttatataggcagaaaagatcaagttgca | Upstream flanking fragments |
| **Prophage2-DOWN-F** | cttgatcttttctgcctatataaaggaagggaaat | Downstream flanking fragments |
| **Prophage2-DOWN-R** | ggccagtgccaagctttccttgcgggtgaccacacc | Downstream flanking fragments |
| **V-B6-2-Pro2-UP-F** | aaagcgatgccagaaagcgc | Δprophage 2 strain verification |
| **V-Pro2-UP-F** | aaaccgcttcgatcctggcg | Δprophage 2 strain verification |
| **V-Pro2-DOWN-R** | acaccaggtgcttgtagcgg | Δprophage 2 strain verification |
| **V-B6-2-Pro2-DOWN-R** | tgtccaaagggttgaggcgg | Δprophage 2 strain verification |
| ***endA-*1-UP-F** | acatgattacgaattcaaagcgcgatagctcgcgtc | Upstream flanking fragments |
| ***endA-*1-UP-R** | aaatatgttttccccccgtttcaaaggctgcgcgg | Upstream flanking fragments |
| ***endA-*1-DOWN-F** | cagcctttgaaacggggggaaaacatatttcaggt | Downstream flanking fragments |
| ***endA-*1-DOWN-R** | ggccagtgccaagcttacaccaacctgggctacggc | Downstream flanking fragments |
| **V-B6-2-*endA-*1-UP-F** | accgctgcgctcaatacttc | Δ*endA-*1 strain verification |
| **V-*endA-*1-UP-F** | aacctggtcaacggctgacg | Δ*endA-*1 strain verification |
| **V-*endA-*1-DOWN-R** | attacaatgcgcggcccaac | Δ*endA-*1 strain verification |
| **V-B6-2-*endA-*1-DOWN-R** | agcctgacgctgatggctac | Δ*endA-*1 strain verification |
| ***endA-*2-UP-F** | acatgattacgaattcaccgttaggggaattctgcg | Upstream flanking fragments |
| ***endA-*2-UP-R** | gcgctggcctgaggagcgcagtcaatcttccttcg | Upstream flanking fragments |
| ***endA-*2-DOWN-F** | gaagattgactgcgctcctcaggccagcgcctgta | Downstream flanking fragments |
| ***endA-*2-DOWN-R** | ggccagtgccaagctttcaagcgcgatggccagtac | Downstream flanking fragments |
| **V-B6-2-*endA-*2-UP-F** | atgggcttcgacctgctcac | Δ*endA-*2 strain verification |
| **V-*endA-*2-UP-F** | aatgtcaccctcgcccatcg | Δ*endA-*2 strain verification |
| **V-*endA-*2-DOWN-R** | tggcccgttatcgccaagcc | Δ*endA-*2 strain verification |
| **V-B6-2-*endA-*2-DOWN-R** | agtacgaagcctaccgcagc | Δ*endA-*2 strain verification |
| **Prophage3-UP-F** | acatgattacgaattcaccagcccggattacctgcc | Upstream flanking fragments |
| **Prophage3-UP-R** | tcggcactcttggcgagggctgggccatattggcg | Upstream flanking fragments |
| **Prophage3-DOWN-F** | atatggcccagccctcgccaagagtgccgaaactc | Downstream flanking fragments |
| **Prophage3-DOWN-R** | ggccagtgccaagctttttcgtgttcggcaatgcgc | Downstream flanking fragments |
| **V-B6-2-Pro3-UP-F** | agtggatgagcattggcaag | Δprophage 3 strain verification |
| **V-Pro3-UP-F** | ttaccctcgcctcttgctgc | Δprophage 3 strain verification |
| **V-Pro3-DOWN-R** | actggtaacccaggtcttgc | Δprophage 3 strain verification |
| **V-B6-2-Pro3-DOWN-R** | tgcagtgtttggccacttcc | Δprophage 3 strain verification |
| **Prophage4-UP-F** | acatgattacgaattcagttcgatgccccggacatc | Upstream flanking fragments |
| **Prophage4-UP-R** | acgacggaggagcacgggcgggctgcgaaggccac | Upstream flanking fragments |
| **Prophage4-DOWN-F** | cttcgcagcccgcccgtgctcctccgtcgtagaca | Downstream flanking fragments |
| **Prophage4-DOWN-R** | ggccagtgccaagcttaaagtcgggttctcacaccc | Downstream flanking fragments |
| **V-B6-2-Pro4-UP-F** | acctctcagacgatacggtg | Δprophage 4 strain verification |
| **V-Pro4-UP-F** | agttcgttgtgatcggcttg | Δprophage 4 strain verification |
| **V-Pro4-DOWN-R** | ttttccccgttcattccccg | Δprophage 4 strain verification |
| **V-B6-2-Pro4-DOWN-R** | tgcgaggaaggtttccacgc | Δprophage 4 strain verification |
| **GI-1-UP-F** | acatgattacgaattcagctcgcggtcgagttcgtc | Upstream flanking fragments |
| **GI-1-UP-R** | atatggccagcatgaccggaggcaagggtgtcgcg | Upstream flanking fragments |
| **GI-1-DOWN-F** | cacccttgcctccggtcatgctggccatatttggc | Downstream flanking fragments |
| **GI-1-DOWN-R** | ggccagtgccaagctttgaccatcggtcccgatgcg | Downstream flanking fragments |
| **V-B6-2-GI-1-UP-F** | agcagcattctgagccggtc | ΔGI-1 strain verification |
| **V-GI-1-UP-F** | atttgtgccacctcaccccg | ΔGI-1 strain verification |
| **V-GI-1-DOWN-R** | tagtgcctgtgacaggtggc | ΔGI-1 strain verification |
| **V-B6-2-GI-1-DOWN-R** | ttcagcgtggcaccgatgag | ΔGI-1 strain verification |
| **GI-2-UP-F** | acatgattacgaattcaatagcgctccatcagcctc | Upstream flanking fragments |
| **GI-2-UP-R** | cggtagggtgttgccccactttgcatctgcccccc | Upstream flanking fragments |
| **GI-2-DOWN-F** | gcagatgcaaagtggggcaacaccctaccggaaag | Downstream flanking fragments |
| **GI-2-DOWN-R** | ggccagtgccaagctttgacacaacgcgcaaacccc | Downstream flanking fragments |
| **V-B6-2-GI-2-UP-F** | atggctgtccgttttgcacc | ΔGI-2 strain verification |
| **V-GI-2-UP-F** | agacccgttttggcatcacc | ΔGI-2 strain verification |
| **V-GI-2-DOWN-R** | taggtgagagatcgggcggc | ΔGI-2 strain verification |
| **V-B6-2-GI-2-DOWN-R** | atgccctggcaccaatcctg | ΔGI-2 strain verification |
| ***vdh*-UP-F** | catgattacgaattcacgtgaagttggccgccaac | Upstream flanking fragments |
| ***vdh*-UP-R** | agatctgcccctggttcgaccgtggcatccaggtc | Upstream flanking fragments |
| ***vdh*-DOWN-F** | ggatgccacggtcgaaccaggggcagatctgcatg | Downstream flanking fragments |
| ***vdh*-DOWN-R** | gccagtgccaagctttaccgaagctgccatagccg | Downstream flanking fragments |
| **V-B6-2-*vdh*-UP-F** | aggcgctgatctgttgcagg | Δ*vdh* strain verification |
| **V-*vdh*-UP-F** | acctcaagccagccctgctc | Δ*vdh* strain verification |
| **V-*vdh*-DOWN-R** | tacggctgtccaccacaagg | Δ*vdh* strain verification |
| **V-B6-2-*vdh*-DOWN-R** | ttgtacatcctgccgccgcc | Δ*vdh* strain verification |
| **pK18mob*sacB*-F** | agctggcacgacaggtttcc | Deletion vector verification |
| **pK18mob*sacB*-R** | ggctgcgcaactgttgggaa | Deletion vector verification |

**
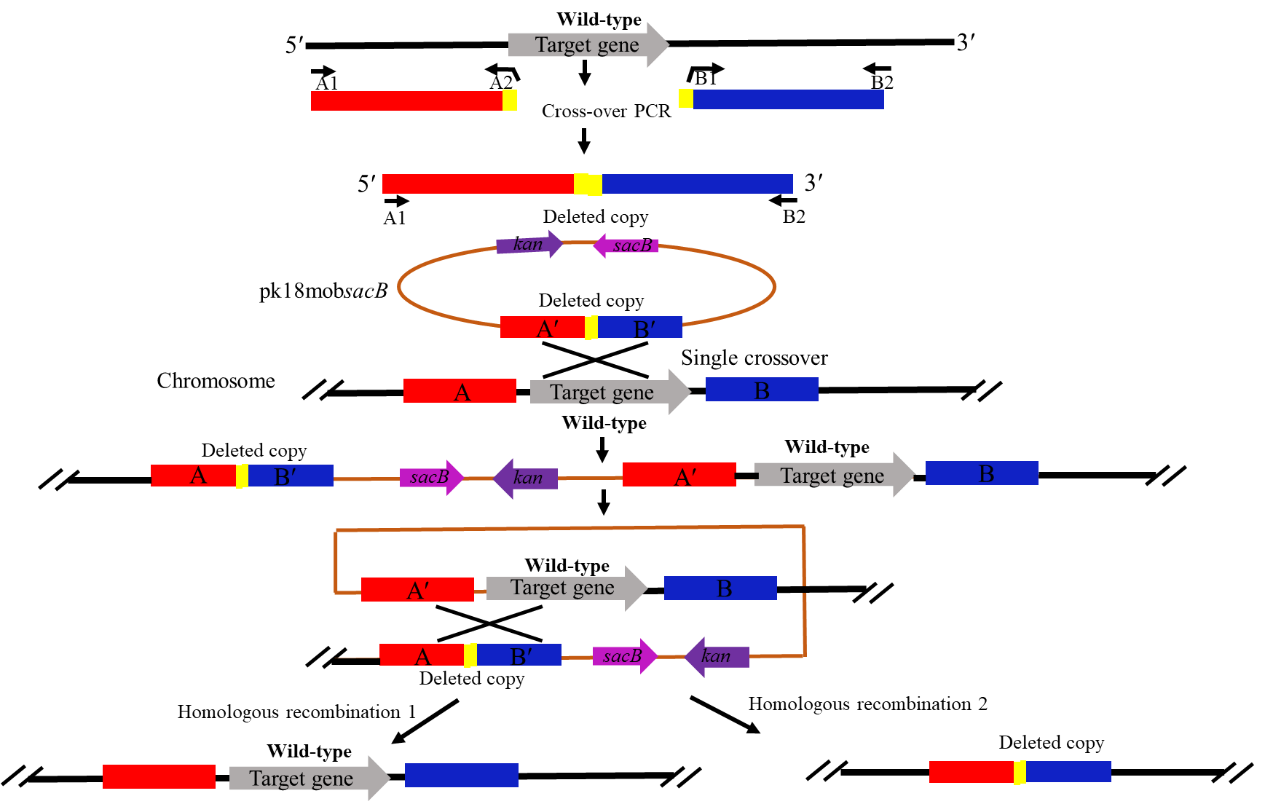
**

Fig. S1 Traceless knockout diagram.


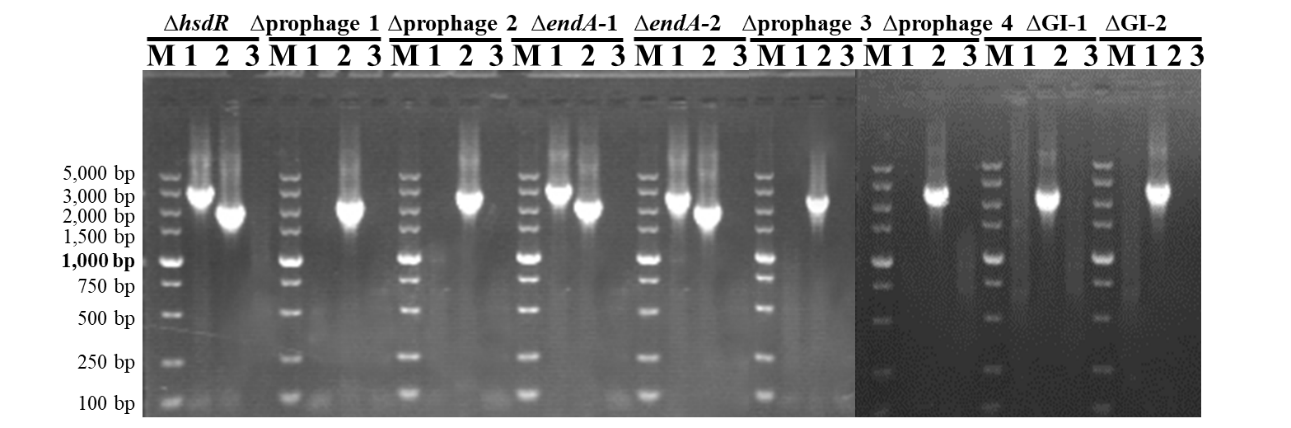


Fig. S2 Confirmation of the deletions by electrophoresis. Lanes: M, DNA marker; 1, *P. putida* B6-2 genomic DNA used as the template; 2, *P. putida* BGR4 genomic DNA used as the template; 3, ddH_2_O used as the template.


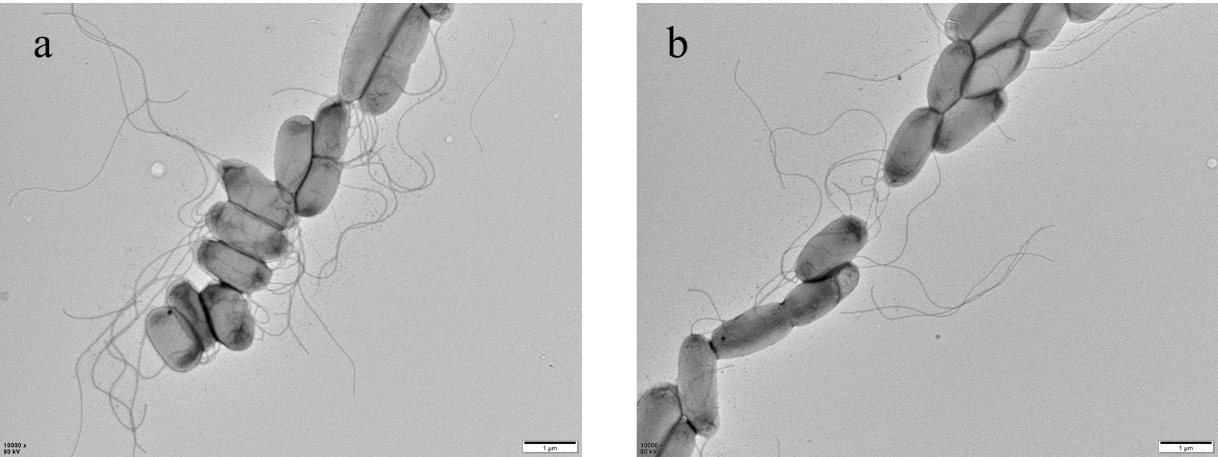


Fig. S3 Transmission electron microscope images of *P. putida* B6-2 **(a)** and *P. putida* BGR4 **(b)** (15,000 ×).


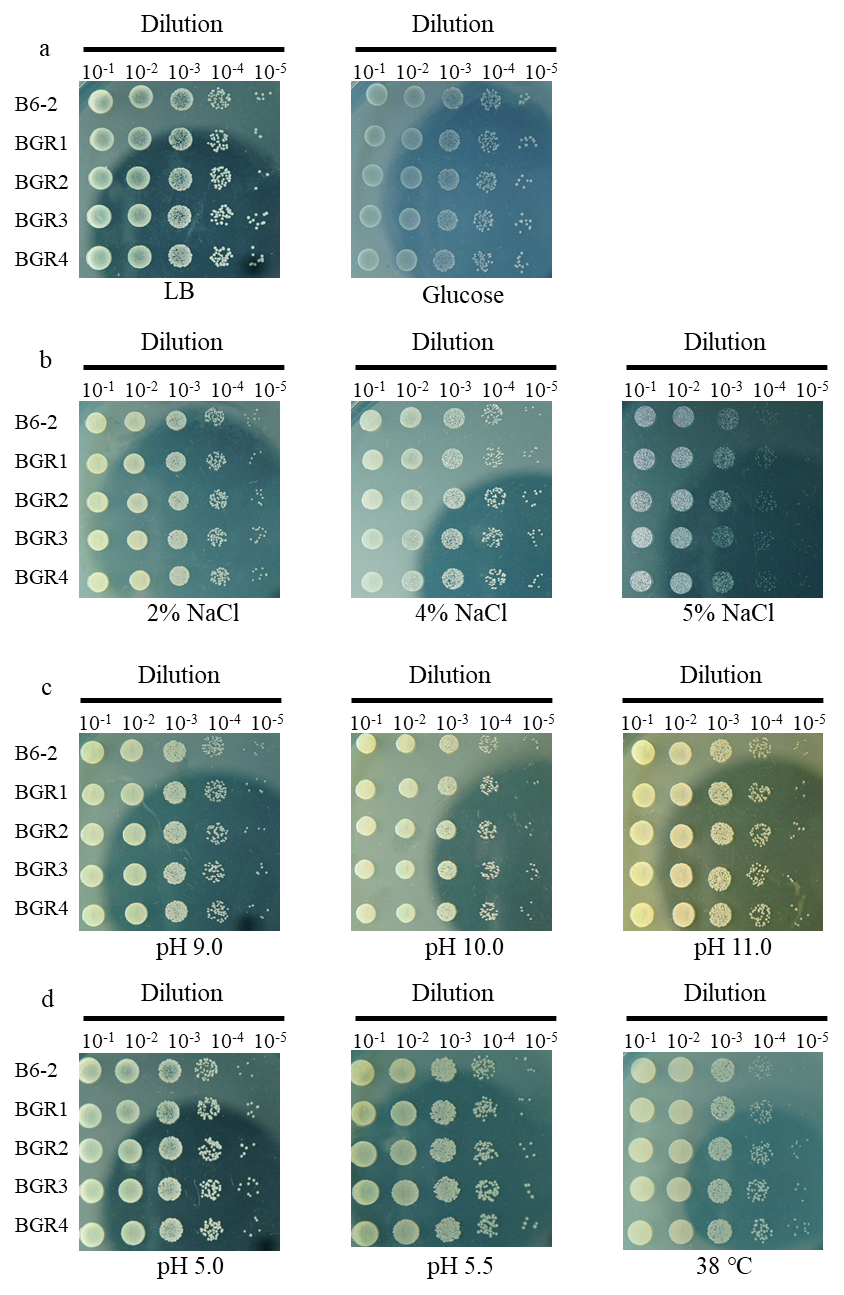


Fig. S4 Drop assays with different carbon sources or under different stress conditions. **(a)** Control plate (plain LB), MSM plate supplemented with 2 g/L glucose. **(b)** LB plates supplemented with 2% (w/v), 4% (w/v) or 5% (w/v) NaCl. **(c)** LB plates at a pH of 9.0, 10.0 or 11.0. **(d)** LB plates at a pH of 5.0 or 5.5 or LB plates cultured at 38 °C.


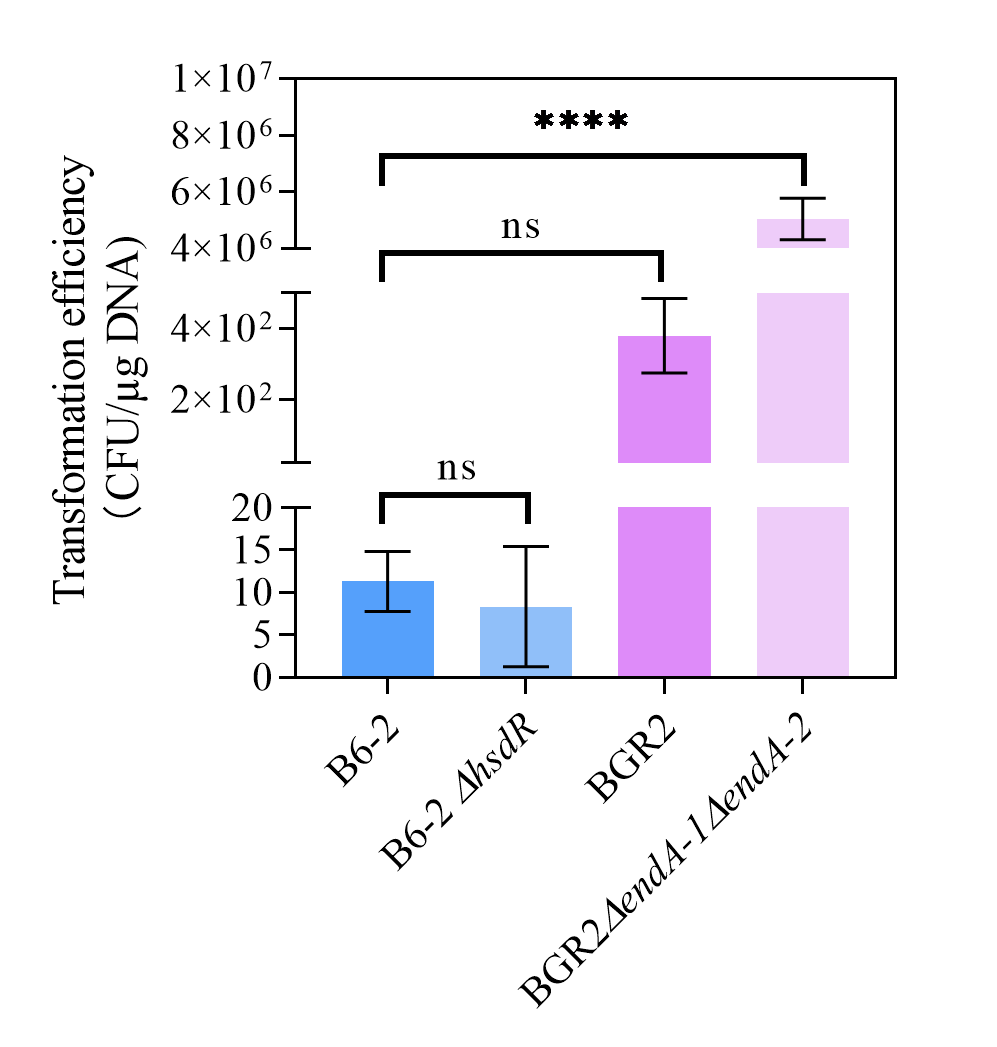


Fig. S5 Transformation efficiencies for the pBBR1MCS2 plasmid of *P. putida* B6-2 and the genome-streamlined strains. One-way ANOVA was performed for statistical analysis. ns: no significant difference, *: *P* <  0.05, **: *P*  <  0.01, ***: *P*  <  0.001, ****: *P*  <  0.0001.

**
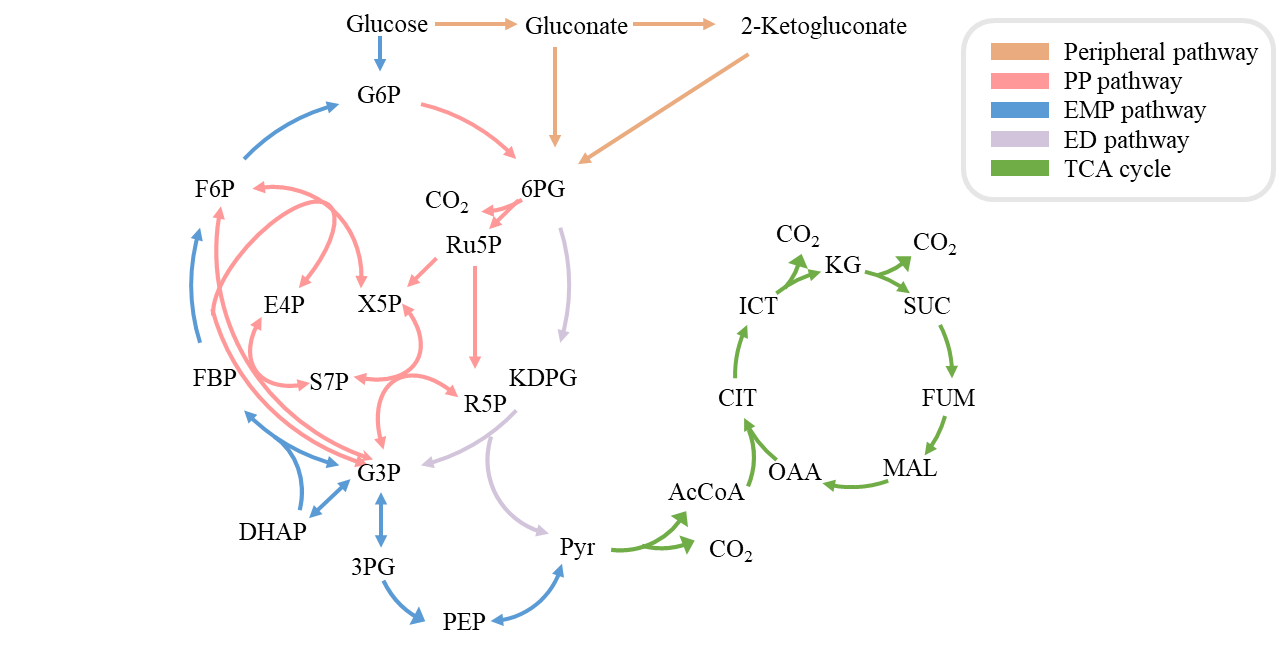
**

Fig. S6 Central carbon metabolism pathways. The central carbon metabolism pathways included the peripheral pathway, the pentose phosphate (PP) pathway, the Embden–Meyerhof–Parnas (EMP) pathway, the Entner-Doudoroff (ED) pathway and the TCA cycle. Abbreviations: G6P, glucose-6-phosphate; F6P, fructose-6-phosphate; FBP, fructose-1,6-bisphosphate; DHAP, dihydroxyacetone phosphate; 6PG, 6-phosphogluconate; KDPG, 2-keto-3-deoxy-6-phosphogluconate; Ru5P, ribulose-5-phosphate; R5P, ribose-5-phosphate; X5P, xylulose-5-phosphate; S7P, sedoheptulose-7-phosphate; E4P, erythrose-4-phosphate; G3P, glyceraldehyde-3-phosphate; 3PG, 3-phosphoglycerate; 2PG, 2-phosphoglycerate; PEP, phosphoenolpyruvate; Pyr, pyruvate; AcCoA, acetyl-coenzyme A; OAA, oxaloacetate; CIT, citrate; ICT, isocitrate; KG: 2-Oxo-glutarate; SUC, succinate; FUM, fumarate; MAL, malate.


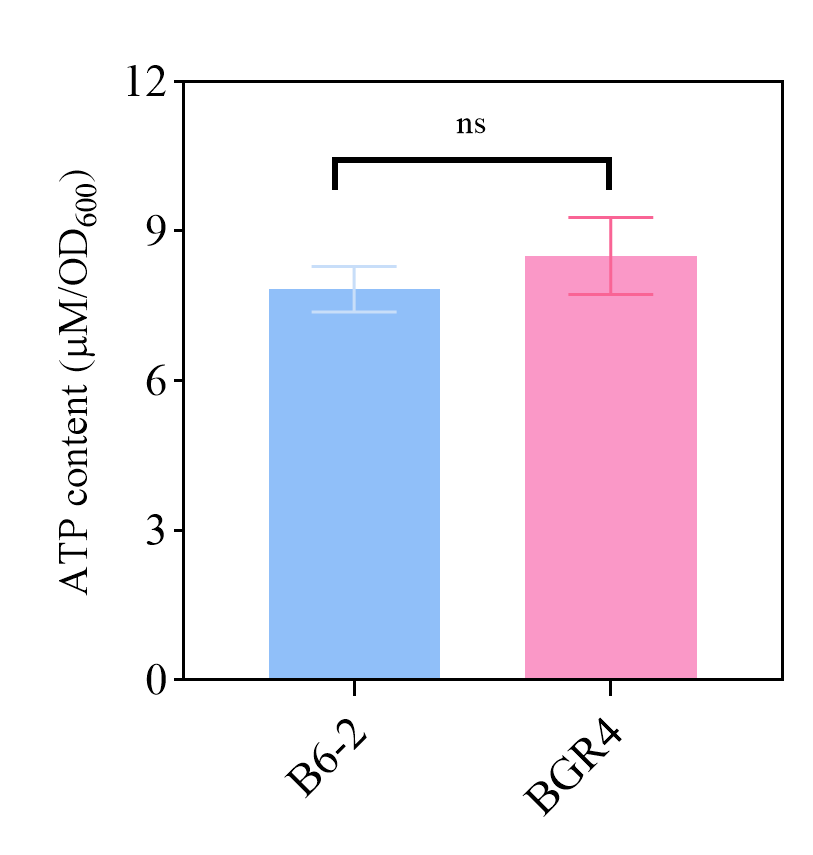


Fig. S7 The ATP content of *P. putida* B6-2 and *P. putida* BGR4. Two-tailed Student’s t tests were performed for statistical analysis. ns: no significant difference.


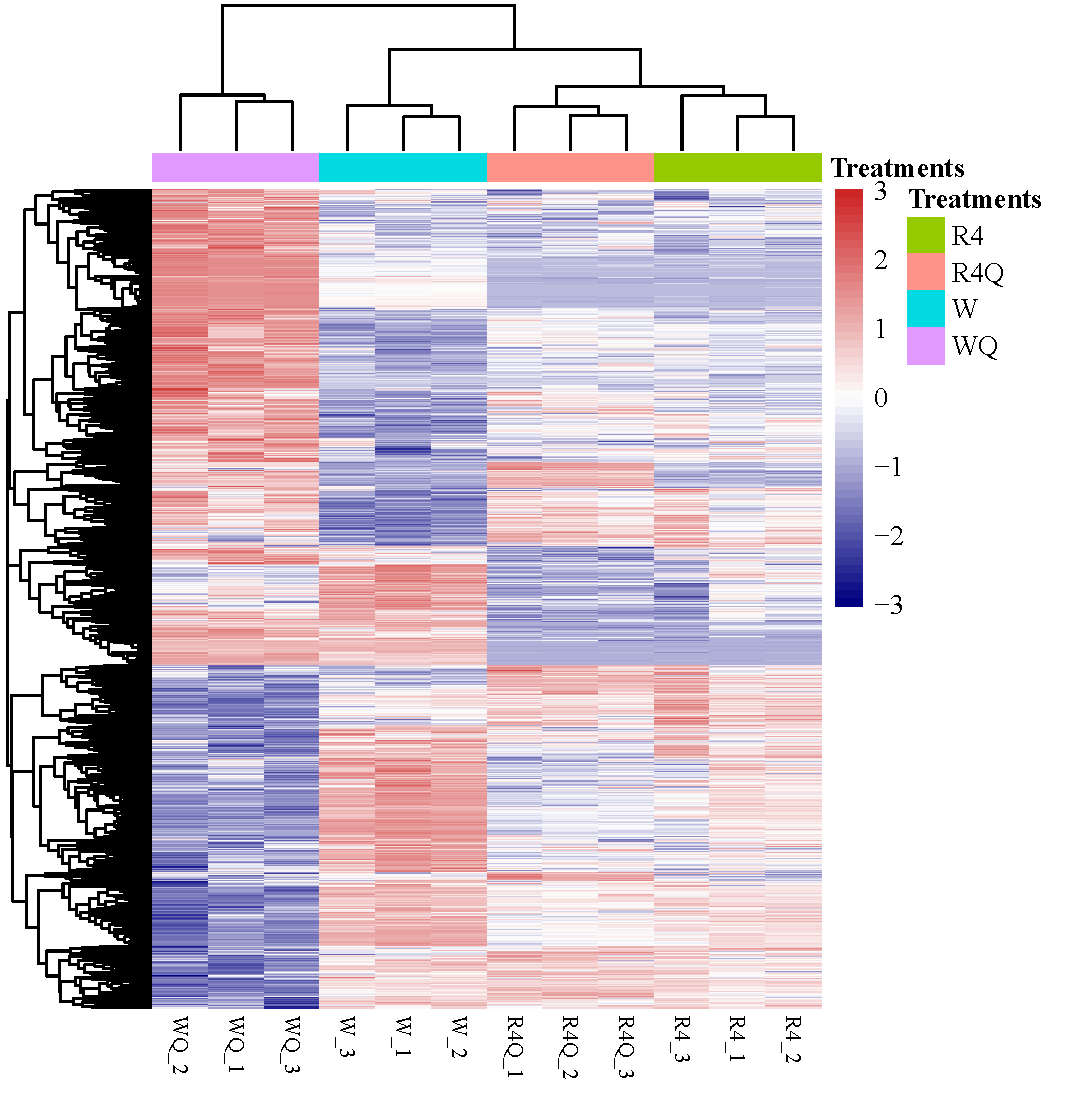


Fig. S8 Cluster analysis of DEGs between the control group (W vs. R4) and the treatment group (WQ vs. R4Q). W, *P. putida* B6-2 cultured in LB medium; R4, *P. putida* BGR4 cultured in LB medium. WQ, *P. putida* B6-2 cultured in LB medium supplemented with 0.6 mM 4-NQO; R4Q, *P. putida* BGR4 cultured in LB medium supplemented with 0.6 mM 4-NQO.


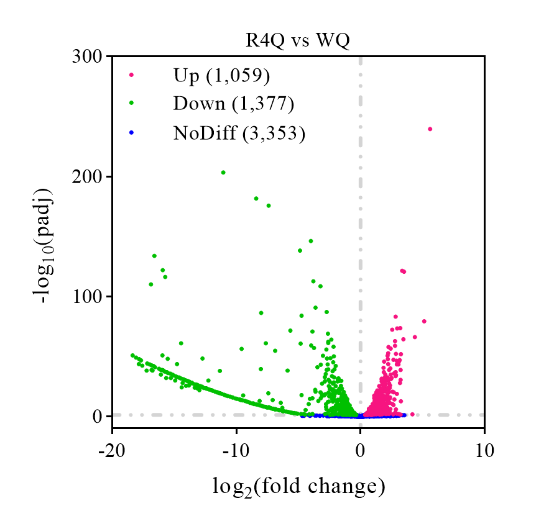


Fig. S9 Volcano map analysis of DEGs in the treatment group (WQ vs. R4Q). WQ, *P. putida* B6-2 cultured in LB medium supplemented with 0.6 mM 4-NQO; R4Q, *P. putida* BGR4 cultured in LB medium supplemented with 0.6 mM 4-NQO.

**
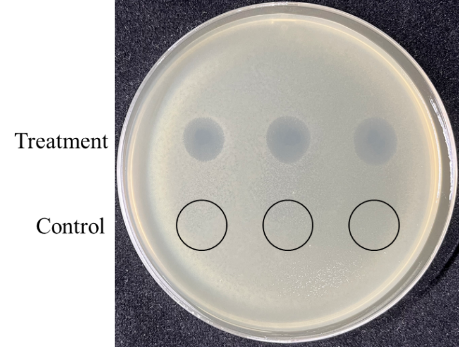
**

Fig. S10 Phage infection test. The treatment sample was the supernatant of an overnight culture of *P. putida* B6-2 after treatment with mitomycin C and chloroform. The control sample was LB medium supplemented with the same concentration of mitomycin C and chloroform. The details of the preparation of the treatment and control samples were the same as those for the phage infection test.


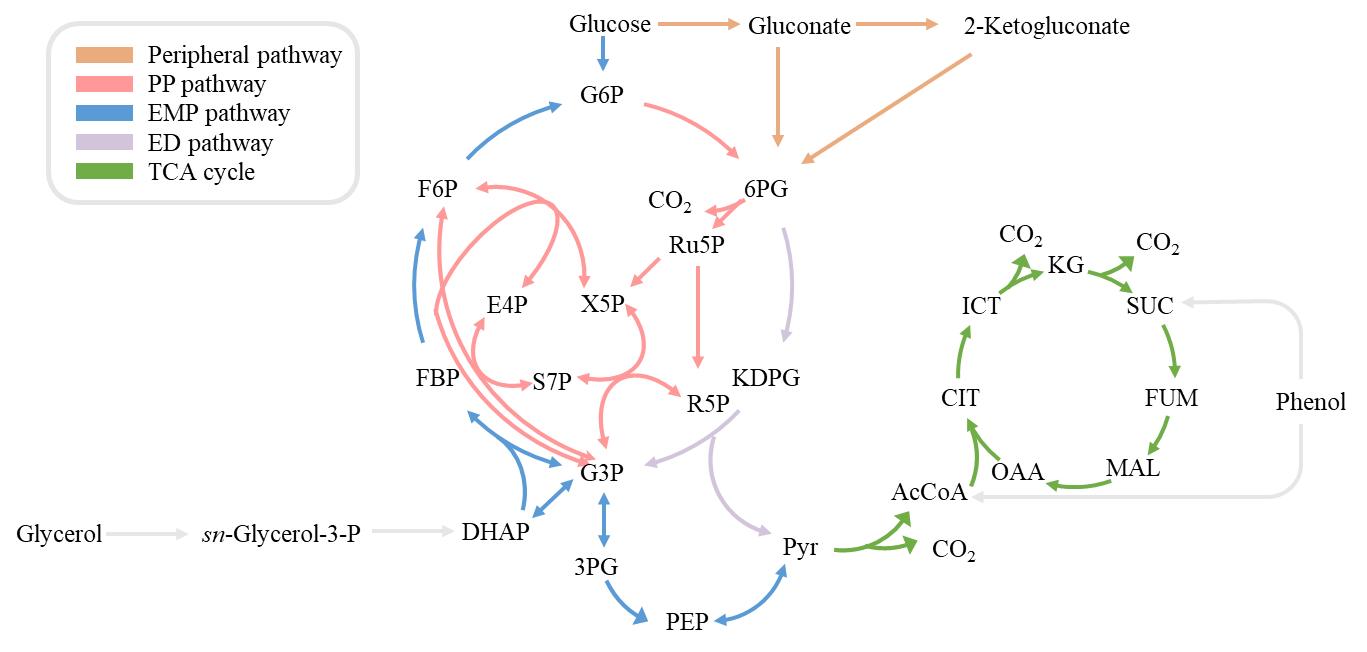


Fig. S11 Metabolism of phenol and glycerol in the *P. putida* strains.
