# Supplementary material for: Genome streamlining of Pseudomonas putida B6-2 for bioremediation
Source: mSystems. 2024 Nov 12;9(12):e00845-24. doi: 10.1128/msystems.00845-24 (PMC11658094; doi:10.1128/msystems.00845-24)
Supplement: Legends — Supplemental material legends. [file msystems.00845-24-s0009.docx]

**Supplemental material legend:**

**Additional Files 1:**

Table S1 Comparison of the degradation characteristics of *P. putida* KT2440 and *P. putida* B6-2.

Table S2 Gene annotation of the deleted regions of *P. putida* B6-2.

Table S3 Detailed information about the GEMs.

Table S4 Results of the GEN III Micro-Plate™ test and the wild_type_B6-2 model prediction.

Note: The symbol '-' indicates no growth in the GEN III Micro-Plate™ test, while the symbol '/' indicates no growth in the wild_type_B6-2 model-predicted results. The maximum uptake rate of the substrates in the wild_type_B6-2 model was defined as 10 mmol/g/h.

Table S5 The utilization capabilities of *P. putida* B6-2 for 7 carbon sources predicted by the wild_type_B6-2 model.

Note: The symbol '/' indicates no growth in the wild_type_B6-2 model-predicted results. The maximum uptake rate of the substrates in the wild_type_B6-2 model was defined as 10 mmol/g/h.

Table S6 GEM-predicted results for the MGRs (h^-1^) on LB, 28 positive carbon sources and phenol.

Note: The maximum uptake rate of the substrates of LB in the GEMs was defined as 1 mmol/g/h, and that of other substrates was defined as 10 mmol/g/h.

Table S7 Sequences of primers used in this study.

Fig. S1 Traceless knockout diagram.

Fig. S2 Confirmation of the deletions by electrophoresis. Lanes: M, DNA marker; 1, *P. putida* B6-2 genomic DNA used as the template; 2, *P. putida* BGR4 genomic DNA used as the template; 3, ddH_2_O used as the template.

Fig. S3 Transmission electron microscope images of *P. putida* B6-2 (a) and *P. putida* BGR4 (b) (15,000 ×).

Fig. S4 Drop assays with different carbon sources or under different stress conditions. (a) Control plate (plain LB), MSM plate supplemented with 2 g/L glucose. (b) LB plates supplemented with 2% (w/v), 4% (w/v) or 5% (w/v) NaCl. (c) LB plates at a pH of 9.0, 10.0 or 11.0. (d) LB plates at a pH of 5.0 or 5.5 or LB plates cultured at 38 °C.

Fig. S5 Transformation efficiencies for the pBBR1MCS2 plasmid of *P. putida* B6-2 and the genome-streamlined strains. One-way ANOVA was performed for statistical analysis. ns: no significant difference, *: *P* <  0.05, **: *P*  <  0.01, ***: *P*  <  0.001, ****: *P*  <  0.0001.

Fig. S6 Central carbon metabolism pathways. The central carbon metabolism pathways included the peripheral pathway, the pentose phosphate (PP) pathway, the Embden–Meyerhof–Parnas (EMP) pathway, the Entner-Doudoroff (ED) pathway and the TCA cycle. Abbreviations: G6P, glucose-6-phosphate; F6P, fructose-6-phosphate; FBP, fructose-1,6-bisphosphate; DHAP, dihydroxyacetone phosphate; 6PG, 6-phosphogluconate; KDPG, 2-keto-3-deoxy-6-phosphogluconate; Ru5P, ribulose-5-phosphate; R5P, ribose-5-phosphate; X5P, xylulose-5-phosphate; S7P, sedoheptulose-7-phosphate; E4P, erythrose-4-phosphate; G3P, glyceraldehyde-3-phosphate; 3PG, 3-phosphoglycerate; 2PG, 2-phosphoglycerate; PEP, phosphoenolpyruvate; Pyr, pyruvate; AcCoA, acetyl-coenzyme A; OAA, oxaloacetate; CIT, citrate; ICT, isocitrate; KG: 2-Oxo-glutarate; SUC, succinate; FUM, fumarate; MAL, malate.

Fig. S7 The ATP content of *P. putida* B6-2 and *P. putida* BGR4. Two-tailed Student’s t tests were performed for statistical analysis. ns: no significant difference.

Fig. S8 Cluster analysis of DEGs between the control group (W vs. R4) and the treatment group (WQ vs. R4Q). W, *P. putida* B6-2 cultured in LB medium; R4, *P. putida* BGR4 cultured in LB medium. WQ, *P. putida* B6-2 cultured in LB medium supplemented with 0.6 mM 4-NQO; R4Q, *P. putida* BGR4 cultured in LB medium supplemented with 0.6 mM 4-NQO.

Fig. S9 Volcano map analysis of DEGs in the treatment group (WQ vs. R4Q). WQ, *P. putida* B6-2 cultured in LB medium supplemented with 0.6 mM 4-NQO; R4Q, *P. putida* BGR4 cultured in LB medium supplemented with 0.6 mM 4-NQO.

Fig. S10 Phage infection test. The treatment sample was the supernatant of an overnight culture of *P. putida* B6-2 after treatment with mitomycin C and chloroform. The control sample was LB medium supplemented with the same concentration of mitomycin C and chloroform. The details of the preparation of the treatment and control samples were the same as those for the phage infection test.

Fig. S11 Metabolism of phenol and glycerol in the *P. putida* strains.

**Additional Files 2:**

Detailed information and comparisons of the reactions of the 5 models.

**Additional Files 3:**

Detailed information and comparisons of the metabolites of the 5 models.

**Additional Files 4:**

The Memote report of model wild_type_B6-2.

**Additional Files 5:**

The Memote report of model BGR1.

**Additional Files 6:**

The Memote report of model BGR2.

**Additional Files 7:**

The Memote report of model BGR3.

**Additional Files 8:**

The Memote report of model BGR4.
